# Microalgae-based oral microcarriers for gut microbiota homeostasis and intestinal protection in cancer radiotherapy

**DOI:** 10.1038/s41467-022-28744-4

**Published:** 2022-03-17

**Authors:** Dongxiao Zhang, Danni Zhong, Jiang Ouyang, Jian He, Yuchen Qi, Wei Chen, Xingcai Zhang, Wei Tao, Min Zhou

**Affiliations:** 1 Department of Respiratory and Critical Care Medicine, The Fourth Affiliated Hospital, Zhejiang University School of Medicine, 322000 Yiwu, China; 2 Institute of Translational Medicine, Zhejiang University, 310029 Hangzhou, China; 3 Center for Nanomedicine and Department of Anesthesiology, Brigham and Women’s Hospital, Harvard Medical School, Boston, 02115 MA USA; 4 School of Engineering and Applied Sciences, Harvard University, Cambridge, MA 02138 USA; 5 School of Engineering, Massachusetts Institute of Technology, Cambridge, MA 02139 USA; 6 State Key Laboratory of Modern Optical Instrumentations, Zhejiang University, 310058 Hangzhou, China; 7 Cancer Center, Zhejiang University, 310058 Hangzhou, China

**Keywords:** Radiotherapy, Drug delivery, Biomedical engineering, Drug delivery, Drug delivery

## Abstract

Protecting the whole small intestine from radiation-induced intestinal injury during the radiotherapy of abdominal or pelvic solid tumors remains an unmet clinical need. Amifostine is a promising selective radioprotector for normal tissues. However, its oral application in intestinal radioprotection remains challenging. Herein, we use microalga *Spirulina platensis* as a microcarrier of Amifostine to construct an oral delivery system. The system shows comprehensive drug accumulation and effective radioprotection in the whole small intestine that is significantly superior to free drug and its enteric capsule, preventing the radiation-induced intestine injury and prolonging the survival without influencing the tumor regression. It also shows benefits on the gut microbiota homeostasis and long-term safety. Based on a readily available natural microcarrier, this work presents a convenient oral delivery system to achieve effective radioprotection for the whole small intestine, providing a competitive strategy with great clinical translation potential.

## Abstract (web summary)

Protecting the whole small intestine from radiation-induced intestinal injury during the radiotherapy of abdominal or pelvic solid tumors remains an unmet clinical need. Here the authors use a natural microalga to readily construct an oral delivery system to achieve effective radioprotection for the small intestine.

## Introduction

Radiotherapy has been extensively used for more than half of cancer patients in clinical settings^1^. However, the damage to surrounding healthy tissues by ionizing radiation can cause a variety of side effects. In the radiotherapy of abdominal/pelvic solid tumors (e.g., pancreatic, prostate, colorectal cancer, etc.), the small intestine with a high radiation sensitivity and large organ volume, is a common site of radiation-induced injury. Intestinal damage caused by intensive radiotherapy can lead to gastrointestinal dysfunction, such as nausea, diarrhea, vomiting, bleeding, infection, perforation, and even death^2^. Therefore, the prevention of radiation-induced intestinal injury is highly desired in radiotherapy. In general, the injured parts of the small intestine are unpredictable among the population since the radiation field varies with the individuals’ tumor sites^3^. This makes comprehensive protection for the whole small intestine an important concern. Another priority is to achieve selective protection on normal tissue without weakening the radiation’s therapeutic effect on tumors. These aspects have been neither focused nor well addressed previously, although many potential radioprotectants are being studied^4^.

Amifostine (AMF) is a selective normal tissue radioprotectant approved by the U.S. Food and Drug Administration (FDA) that needs to be intravenously administered before radiotherapy^4,5^. Although AMF has been used to reduce radiation-induced xerostomia^5^, there are still some challenges to be addressed in its use in the whole intestinal radioprotection. First, intravenous administration is not an ideal delivery route to achieve intestinal distribution due to the rapid clearance of AMF from blood circulation^6^. Although high doses of AMF may improve its accumulation in the intestine, the systemic adverse effects, such as hypotension, nausea, and vomiting would limit its further biomedical applications^7^. Moreover, as AMF can be converted into its inactive metabolites by strong acid^8,9^, the destruction of AMF by gastric acid may reduce the distribution of active drugs entering the intestine. Besides, soluble small-molecule drugs tend to be easily absorbed into circulation in the proximal small intestine^10,11^, which may lead to a relatively insufficient accumulation in the whole small intestine. Oral drug delivery platforms hold great promise in improving drug distribution, bioavailability and efficacy^12,13^. Therefore, a suitable oral delivery strategy that can address these problems would be beneficial to exert the radioprotection of AMF on the small intestine.

*Spirulina platensis* (*S. platensis*, SP), a natural microalga with a length of 200–500 μm and three-dimensional (3-D) helical shape, is an edible microorganism that has been mass-produced and developed into dietary supplementations due to its richness in multiple nutrients^14^. The oral administration of this digestible microalga has shown antioxidative, anti-inflammatory effects, and regulation of intestinal microbiota^15^, which would be beneficial to the prevention and treatment of many intestinal diseases^16,17^. Importantly, SP is a versatile microcarrier for small molecule drugs^18,19^ and showed potential for drug delivery in intestinal diseases in our previous work^20^. On this basis, this work aims to focus on constructing an orally delivered system with the translational potential to address the specific issue of gut microbiota homeostasis and whole intestinal protection in cancer radiotherapy.

Herein, we used SP as the natural microcarrier of AMF to facilely construct an oral drug delivery system, SP@AMF, which could be orally administered for the radioprotection of the whole small intestine. In this system, AMF can be slowly released into simulated intestinal fluid and prolong its protection on intestinal cells in vitro. With micron size and progressively degradation in vivo, it can form a prolonged and comprehensive intestinal distribution. Importantly, compared with AMF and its enteric capsule (Cap@AMF), SP@AMF shows a better radioprotective effect on all parts of the small intestine. Moreover, the usage of SP@AMF does not protect orthotopic colorectal tumors from radiotherapy, showing selective protection on the normal intestine and improvement in survival time. Besides, the use of SP facilitates the homeostasis of gut microbiota after radiation and avoids the long-term toxicity of AMF. This work presents a microalgae-based system to overcome the challenges in AMF’s oral delivery for the whole intestinal radioprotection, thus providing an effective orally delivered radioprotectant with translational potential for protecting the normal intestine in cancer radiotherapy.

## Results

### Synthesis and characterization

**Fig. 1:**
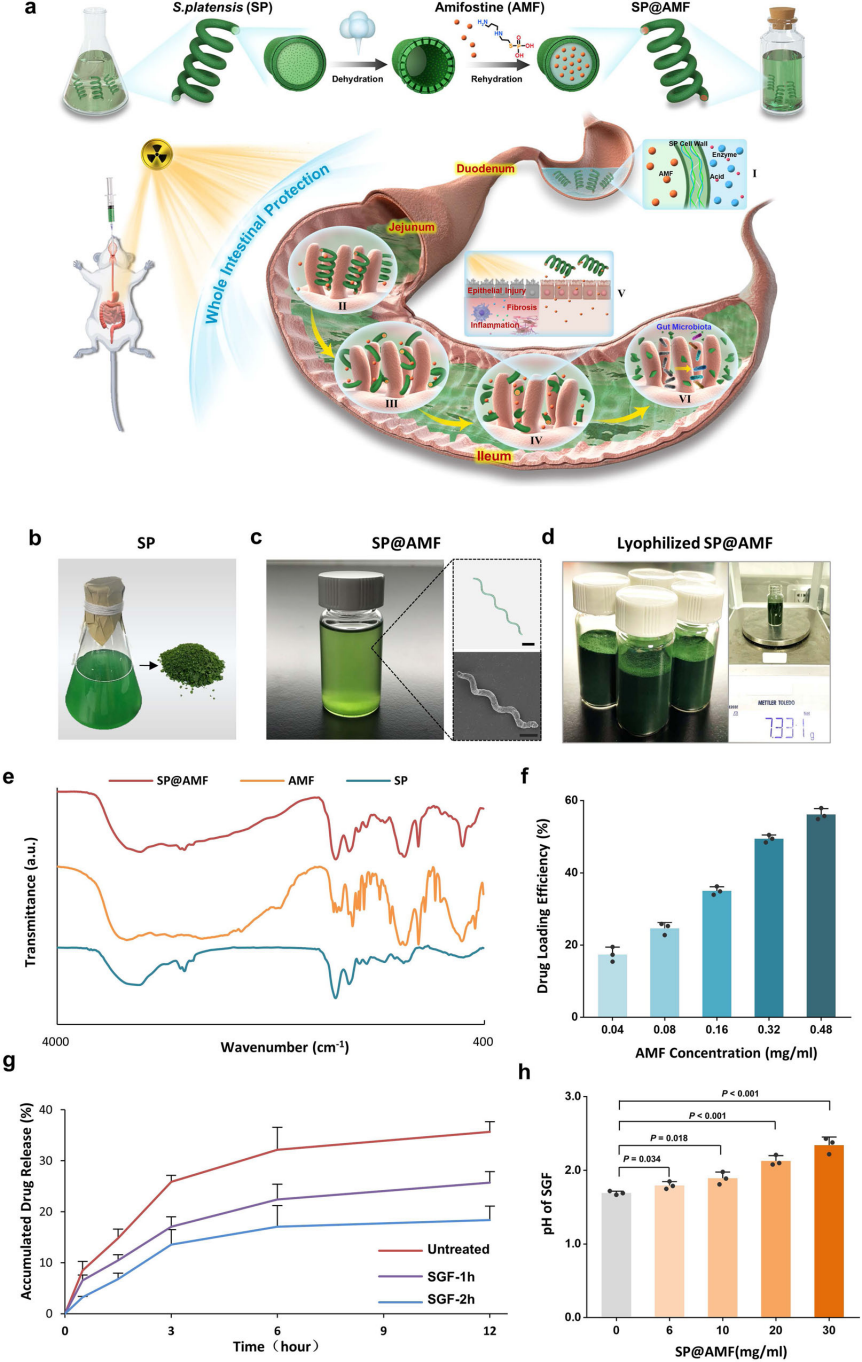
Synthesis and characterization of SP@AMF. **a** Schematic illustration of synthetic protocols and radioprotective mechanisms of SP@AMF. I. SP protects AMF from gastric destruction. II-IV. SP@AMF gradually degrades and slowly releases AMF through the whole small intestine (duodenum, jejunum and ileum). V. SP@AMF protects intestinal tissue from radiation-induced epithelial injury, inflammation, and fibrosis. VI. SP@AMF maintains the health of gut microbiota. **b** SP cultivated in medium and its lyophilized powder. **c** PBS suspension of the prepared SP@AMF and its bright-field microscope and SEM images. Scale bar = 20 µm. **d** Lyophilized powder of the mass prepared SP@AMF. The net weight of SP@AMF in the bottle is 7.331 grams. **e** Fourier transform infrared (FTIR) spectra of SP, AMF, and SP@AMF. **f** Drug loading efficiency (DLE) under various concentrations of AMF loading solution (*n* = 3 independent experiments). The data show means + SD. **g** Release profiles of AMF from SP@AMF in simulated intestinal fluid (SIF) after being treated by simulated gastric fluid (SGF) for 1 and 2 h. Untreated SP@AMF was used as control (*n* = 3 independent experiments). The data show means + SD. **h** pH values of the SGF supernatant containing different concentrations of SP@AMF (*n* = 3 independent experiments). The data show means + SD. *P* was calculated using two-tailed *t*-test.

In this study, a facile dehydration–rehydration synthetic strategy was employed to obtain a high drug loading efficacy of AMF in SP, in which lyophilization was used for the dehydration of SP (Fig. 1a, b). Compared with fresh SP, lyophilized SP showed higher drug loading efficiency of AMF (Supplementary Fig. 1). As shown in Fig. 1c and Supplementary Fig. 2, the synthesized SP@AMF kept the helical shape and chlorophyll fluorescence of SP. SP@AMF can be mass prepared and lyophilized for later use (Fig. 1d). Fourier transform infrared (FTIR) spectra (Fig. 1e) confirmed the successful loading of AMF in SP, in which SP@AMF had the main characteristic absorption peaks of both SP and AMF. The optimal AMF loading concentration and loading time under experimental conditions were 0.48 mg/ml and 12 h, respectively, which were determined by testing various AMF loading concentrations (Fig. 1f) and loading time (Supplementary Fig. 4). The SP@AMF pre-treated with simulated gastric fluid (SGF) could slowly release AMF into the simulated intestinal fluid (SIF) (Fig. 1g). Even after the pre-treatment of SGF for 2 h (SGF-2h), SP@AMF kept approximately half of the release capacity compared with the untreated group. After pre-treatment, the drug-releasing became slower. This might be because SP@AMF had already released some drug into SGF during the pretreatment, which reduced the concentration gradient between inside and outside of SP and thus reduced the releasing speed. However, the underlying specific mechanism still needs to be further investigated. Negligible changes in shape and length of most SGF-treated SP cells were observed (Supplementary Fig. 5), demonstrating the resistance of SP against the destruction of SGF. Moreover, SP showed a mild effect on reducing the acidity of SGF in vitro (Fig. 1h), which could alleviate the harsh condition affecting the drug activity.

### In vitro toxicity and radioprotective effect

**Fig. 2:**
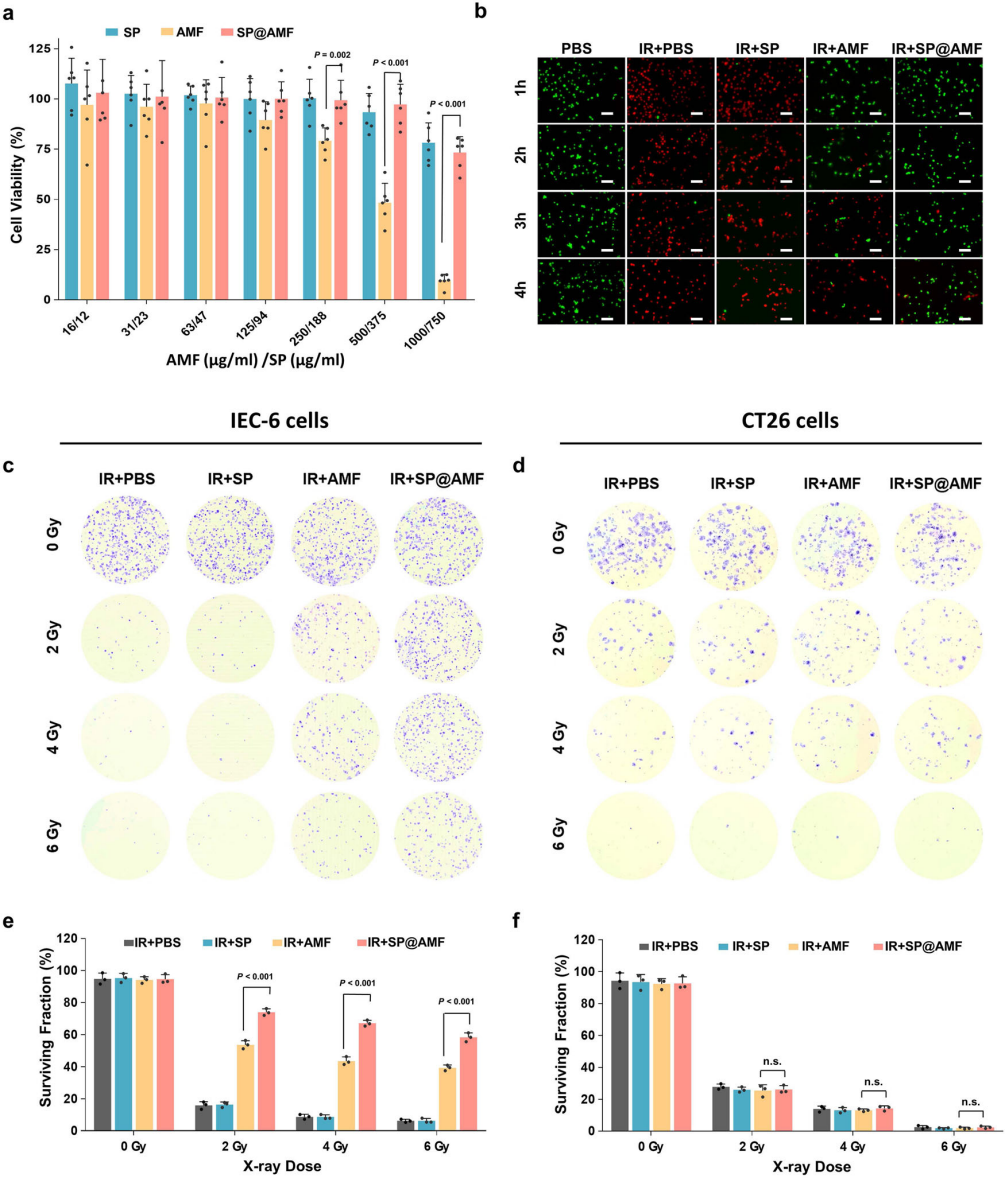
In vitro, SP@AMF shows lower toxicity and more effective protection on normal cells than free AMF. **a** Viabilities of the IEC-6 cells (small intestinal epithelium cells) after incubating with various concentrations of SP, AMF, and SP@AMF for 24 h. The viabilities were determined by an MTT assay kit (*n* = 6 biologically independent cells). The data show means + SD. *P* was calculated using two-tailed *t*-test. **b** Calcein-AM/PI fluorescence images (green, living cell; red, dead cell) of the IEC-6 cells irradiated by 6 Gy X-ray (IR) (except for PBS group) after 1, 2, 3, or 4 h of incubation with the renewed medium in different groups. Scale bar = 100 µm. Experiment was repeated three times independently with similar results. **c**–**f** Crystal violet staining (**c**, **d**) and quantification (**e**, **f**) of the surviving colonies of IEC-6 cells and CT26 cells (colorectal cancer cells) irradiated by 0, 2, 4, and 6 Gy X-ray in different treatment groups (PBS, SP, AMF, and SP@AMF) (*n* = 3 biologically independent cells). The data show means + SD. *P* was calculated using two-tailed *t*-test. n.s. no significance (*P* > 0.05).

To further explore the potential of SP@AMF in biomedical applications, we first employed an MTT assay to study the toxicity of SP@AMF on IEC-6 cells (rat small intestinal epithelium cells) after 24 h of incubation. Notably, the viability of SP@AMF-treated cells was significantly higher than that of AMF-treated cells when AMF concentrations were higher than 125 µg/mL (Fig. 2a).

Subsequently, we evaluated the injury of IEC-6 cells exposed to 6 Gy X-rays at different hours after the renewal of the culture medium containing different materials (Fig. 2b and Supplementary Figs. 6–8). This experiment was designed to simulate the intestinal surface on which digestive fluid is continuously generated (Supplementary Fig. 6). Both double staining (Fig. 2b and Supplementary Fig. 7) and colony formation assays (Supplementary Fig. 8) showed that SP@AMF significantly improved the viability of irradiated cells even 3–4 h after the change of cell medium, while the radiation protection effect of AMF decreased with time. This may be due to the depletion of AMF molecules absorbed by cells over time^21^ when the surrounding AMF-containing medium was removed. By contrast, the SP@AMF remaining around cells slowly released AMF (Fig. 1g), thus providing continuous protection over a longer time. Moreover, the IEC-6 cells exposed to other doses (2 and 4 Gy) of X-ray could also be better protected by SP@AMF than AMF (Fig. 2c–e). By contrast, the colorectal cancer cells (CT26 cells) were not protected by AMF or SP@AMF (Fig. 2d, f), presenting AMF’s selective protective effect on normal cells. One of the main intracellular damages caused by radiation is reactive oxygen species (ROS)-induced DNA double-strand break (DSB)^22,23^. The ROS-scavenging activity of AMF played an important role in preventing DNA damages and subsequent cell disorders^24–26^, although its definite mechanism had not been fully elucidated. Therefore, ROS production and DNA DSB of the irradiated cells were also detected as shown in Supplementary Figs. 9–10, which demonstrated that SP@AMF effectively decreased ROS generation of the irradiated cells and significantly reduced intracellular DSB.

### In vivo biodistribution

**Fig. 3:**
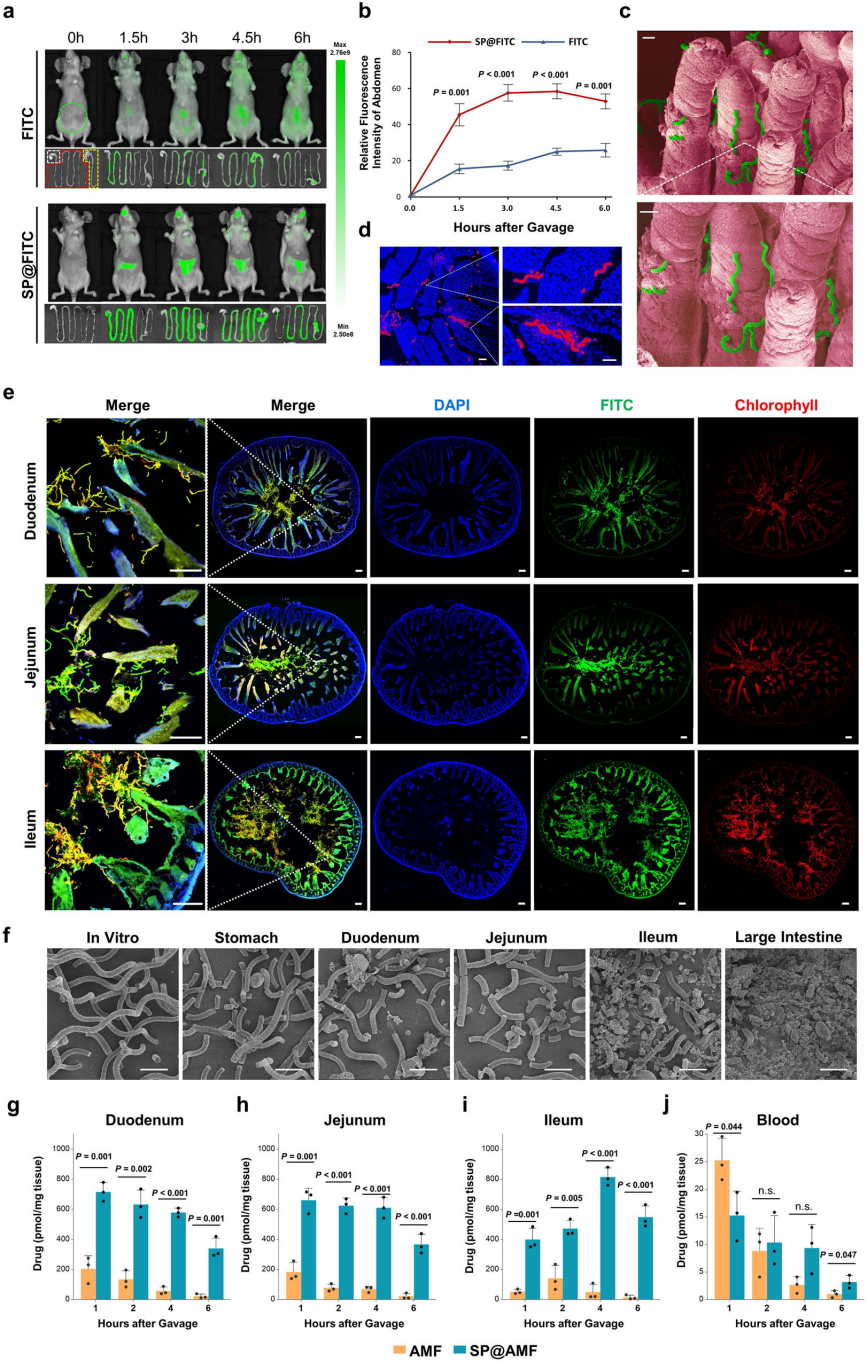
Compared with free drug, SP-based system shows retention among villi, progressive degradation, and extensive drug distribution throughout the small intestine. **a** Fluorescence images of the mice’s body and gastrointestinal tract at 0, 1.5, 3, 4.5, and 6 h after the oral administration of FITC or SP@FITC (with an equal amount of FITC). FITC channel: Ex, 445–490 nm; Em, 515–575 nm. The green dotted circle indicates the analyzed area for the fluorescence intensity quantification (**b**). White, red, and yellow dotted lines indicate the stomach, small intestine, and large intestine, respectively. **b** Quantification of the relative FITC fluorescence intensity in the abdominal area, shown by the ratios to the FITC intensity before gavage (*n* = 3 biologically independent animals). The data show means + SD. *P* between two groups was calculated using two-tailed *t*-test. **c**, **d** SEM (pseudo-color) (**c**) and fluorescence images (**d**) (blue, DAPI; red, chlorophyll) of the materials between the intestinal villi. Scale bar = 20 µm. **e** Fluorescence microscope images (blue, DAPI; green, FITC; red, chlorophyll) of SP@FITC in small intestines (duodenum, jejunum, and ileum) at 4 h after the oral administration of SP@FITC. Scale bar = 100 µm. Experiment was repeated three times independently with similar results. **f** SEM images of SP@FITC in the stomach, small intestines, and large intestines at 4 h after the oral administration of SP@FITC. Scale bar =25 µm. **g**–**j** The combined concentration of AMF and its active metabolite WR-1065 in intestinal tissue of duodenum (**g**), jejunum (**h**), ileum (**i**), and blood (**j**) at 1, 2, 4, and 6 h after AMF or SP@AMF treatment at the dose of 200 mg AMF/kg (*n* = 3 biologically independent animals). The data show means + SD. *P* was calculated using two-tailed *t*-test.

To visualize the drug distribution in the whole intestinal tract, a fluorescent molecule FITC was loaded into SP to construct SP@FITC. As shown in Supplementary Fig. 11, the obtained SP@FITC emit both red fluorescence (from SP, chlorophyll) and green fluorescence (from FITC). In vivo fluorescence images after intragastric administration showed that the FITC intensity in the abdominal area of mice in the SP@FITC group was significantly higher than that in the FITC group (Fig. 3a, b). Fluorescence images of the harvested gastrointestinal tract showed that the SP@FITC group exhibited a more homogeneous fluorescence distribution in the whole intestinal tract compared with the FITC group (Fig. 3a). The quantitative analysis of FITC (Supplementary Fig. 14a–c) in tissue verified that all parts of the small intestine in the SP@FITC group contained more FITC at all time points after gavage, while its FITC concentration in blood (Supplementary Fig. 14d) was lower compared with that of the FITC group.

The fluorescence microscope and SEM images of the harvested small intestine 3–4 h after oral gavage (Fig. 3c–e and Supplementary Fig. 14) showed that a large amount of SP@FITC existed between or on the surface of the intestine villi in all parts of the small intestine (Fig. 3e). Moreover, the SEM and microscope images of the collected gastrointestinal contents (Fig. 3f and Supplementary Fig. 15) showed that SP gradually fragmented from the stomach to the intestines. Importantly, the pharmacokinetics of AMF and its active metabolite, WR-1065, in intestine tissue and blood (Fig. 3g–j) at various of time points after gavage of SP@AMF and AMF was detected by liquid chromatography−mass spectrometry (LC-MS). It showed that the total amount of AMF and WR-1065 in the SP@AMF group was significantly higher than that in the AMF group in all three parts of the small intestine at different times (Fig. 3g–i). On the contrary, the blood concentration in the SP@AMF group was lower or similar than that in the AMF group at 1, 2, 4 h after treatment (Fig. 3j). These results indicated that the carrier, SP, could provide the loaded drug a concentrated and uniform distribution in the whole small intestine, which might be attributed to its intestinal retention and progressive degradation. Furthermore, these results suggested that 4 h before radiotherapy might be the appropriate timing to administrate SP@AMF for enough drug accumulation in all parts of the small intestine, which was finally confirmed by testing the radioprotective effect of SP@AMF in mice (Supplementary Fig. 19).

### Protective effect against early and delayed intestinal radiation injury

**Fig. 4:**
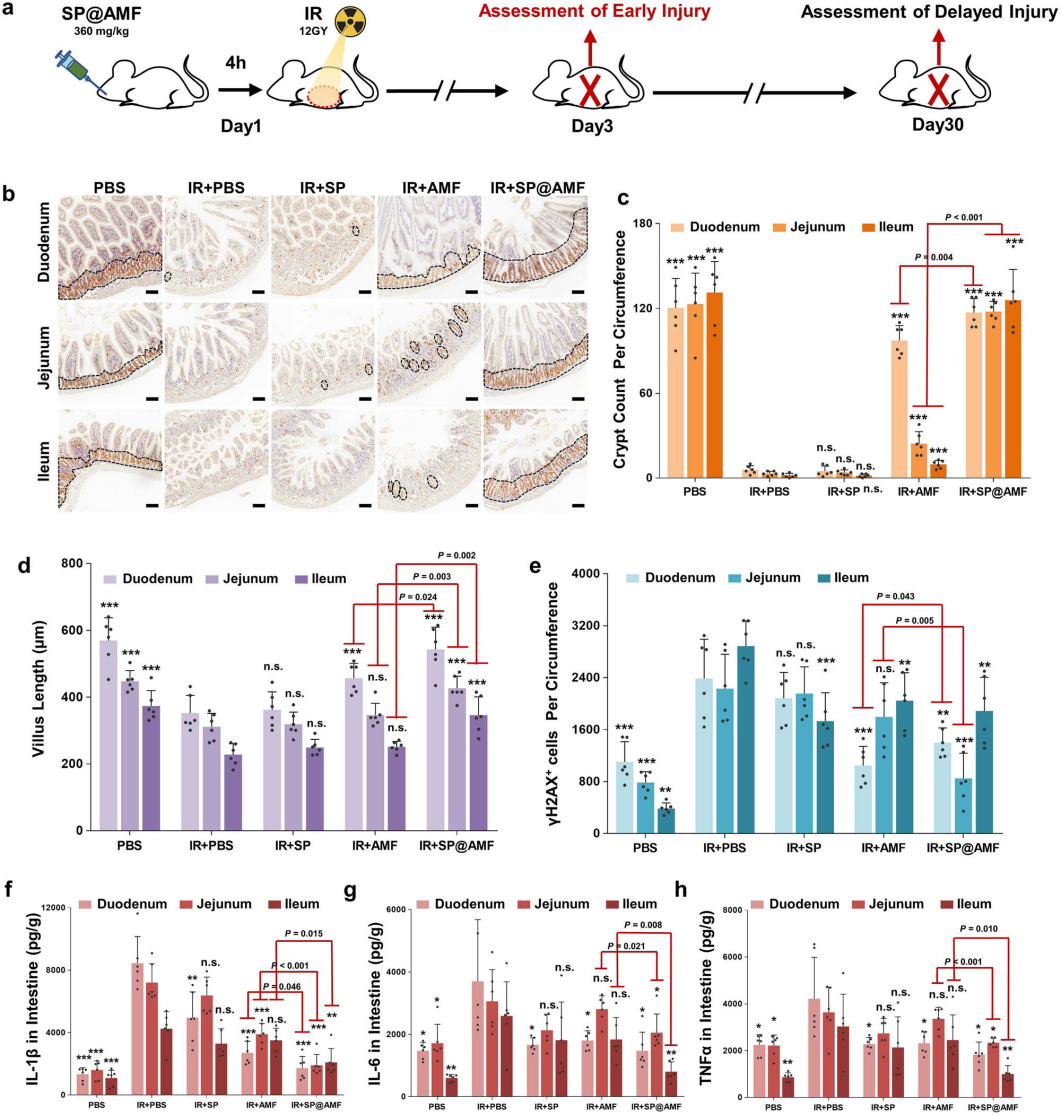
SP@AMF shows comprehensive protective effect overall small intestine against radiation-induced early injury. **a** Schematic illustration of the experiment protocol. **b**, **c** Represented IHC images (**b**) and the quantification (**c**) of the regenerating crypts in the small intestine (duodenum, jejunum, and ileum) stained by Ki67 at day 3 after being treated by sham irradiation + PBS (PBS group), 12 Gy abdominal X-ray (IR) + PBS, IR + SP, IR + AMF, and IR + SP@AMF (*n* = 6 biologically independent animals). The black dotted line indicates the Ki67-stained regenerating crypts. Scale bar =100 µm. Experiment was repeated three times independently with similar results. **d** Quantification of the length of intestinal villi (*n* = 6 biologically independent animals). The represented HE images are shown in Supplementary Fig. 17. **e** Quantification of the γH2AX-positive intestinal cells (*n* = 6 biologically independent animals). The represented IHC images are shown in Supplementary Fig. 18. **f**–**h** Pro-inflammatory cytokines including IL-1β (**f**), IL-6 (**g**), and TNF-α (**h**) in the small intestine tissue (*n* = 6 biologically independent animals). The data show means + SD. *P* was calculated using two-tailed *t*-test. *P versus IR + PBS group (*<0.05, **<0.01, ***<0.001, n.s., no significance).

**Fig. 5:**
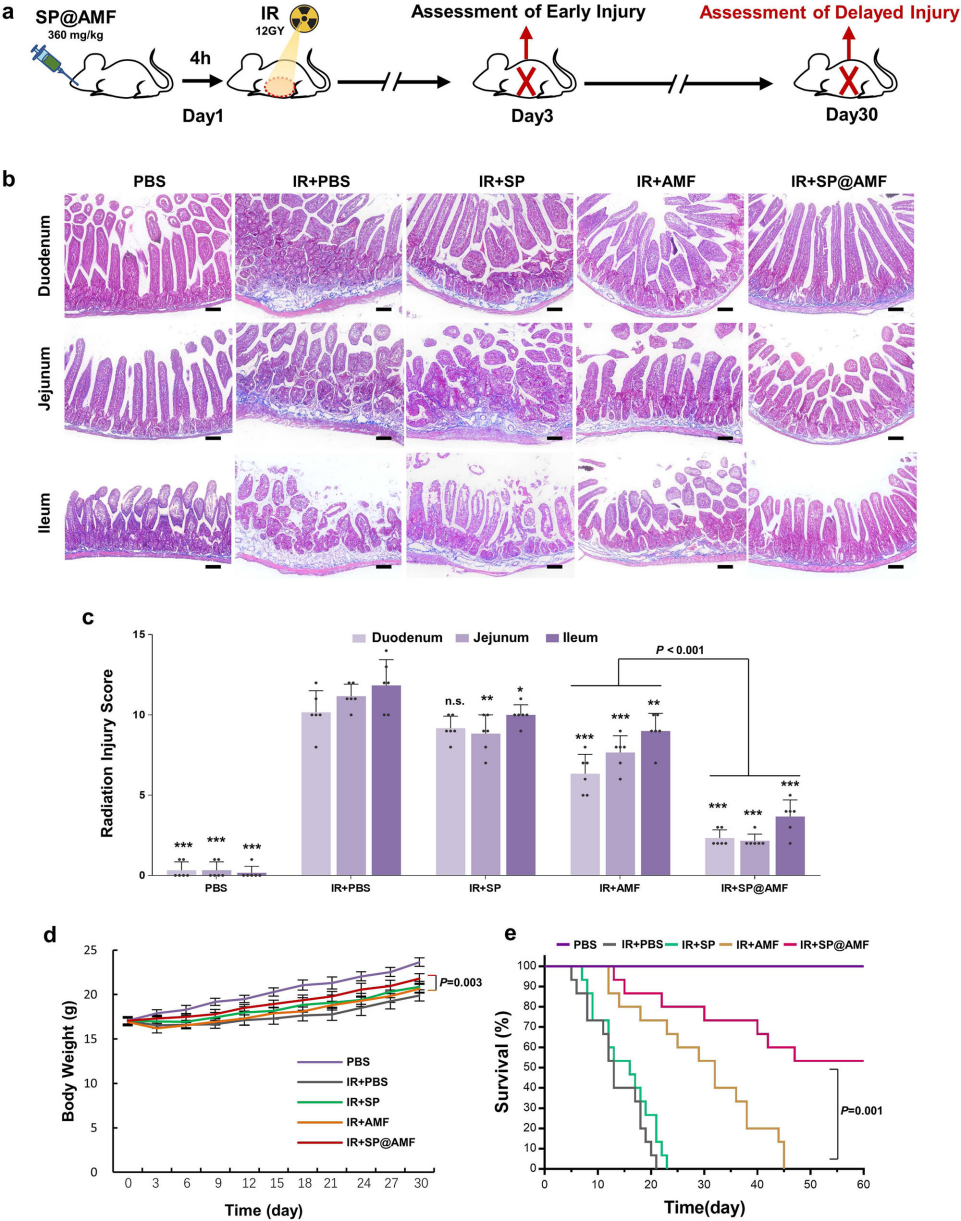
SP@AMF prevents the radiation-induced delayed injury in the whole small intestine, weight loss and death. **a** Schematic illustration of the experiment protocol. **b** Represented IHC images of the small intestine (duodenum, jejunum, and ileum) stained by Masson Trichrome at day 30 after being treated by sham irradiation + PBS (PBS group), 12 Gy abdominal X-ray (IR) + PBS, IR + SP, IR + AMF, and IR + SP@AMF. The blue areas show fibrosis formation. Scale bar =100 µm. Experiment was repeated three times independently with similar results. **c** Scoring of the degree of delayed intestinal radiation (*n* = 6 biologically independent animals). The data show means + SD. *P* was calculated using two-tailed *t*-test. *P versus IR + PBS group (*<0.05, **<0.01, ***<0.001, n.s., no significance). **d** Body weight of the mice (*n* = 6 biologically independent animals). The data show means ± SD. *P* between group IR + AMF and IR + SP@AMF was calculated using two-tailed *t*-test. **e** Survival curves of mice exposed to a fatal dose of abdominal IR (*n* = 15 biologically independent animals). Median survival: PBS, undefined (>60 days); IR + PBS, 13 days; IR + SP, 16 days; IR + AMF, 32 days; IR + SP@AMF, undefined (>60 days). *P* was calculated using Log-rank (Mantel-Cox) test.

In the clinic settings of cancer radiotherapy, radiation can cause various abnormalities of intestinal tissue in all parts of the small intestine, which can be divided into early and delayed injury according to the occurrence time after exposure (Figs. 4a and 5a). Therefore, we tested various representative indicators in the duodenum, jejunum, and ileum in the abdominal irradiation model to evaluate the radioprotective effect of SP@AMF on the whole small intestine (Figs. 4–5 and Supplementary Figs. 20–21).

As shown in Fig. 4b–h and Supplementary Figs. 20–21, some typical abnormalities of radiation-induced early injury, which occurred on 3 days after radiation, were observed in the IR + PBS group, including reduced regenerating crypt cells (Fig. 4b, c), shortened intestinal villi (Fig. 4d and Supplementary Fig. 20), damaged DNA in cells (Fig. 4e and Supplementary Fig. 21) and increased inflammatory factors (Fig. 4f–h). The results also showed that the appearances of injury in the IR + SP@AMF group were significantly less in all three parts of the small intestine. By contrast, in the IR + AMF group, only the proximal (duodenum and jejunum) lesions were mildly prevented, while the distal (ileum) lesions remained severe. Compared with AMF, SP@AMF showed a better protective effect against early injury throughout the whole small intestine. As shown in Fig. 5b, c, the use of SP@AMF also effectively prevented the small intestine from delayed pathologic injury including fibrosis, vascular sclerosis, and atrophy of the mucosa, which was evaluated by the radiation injury score^27^ (Supplementary Table 1). Moreover, SP@AMF significantly improved weight gain (Fig. 5d) and survival (at lethal radiation doses) in mice (Fig. 5e), while AMF only provided less effective protection.

**Fig. 6:**
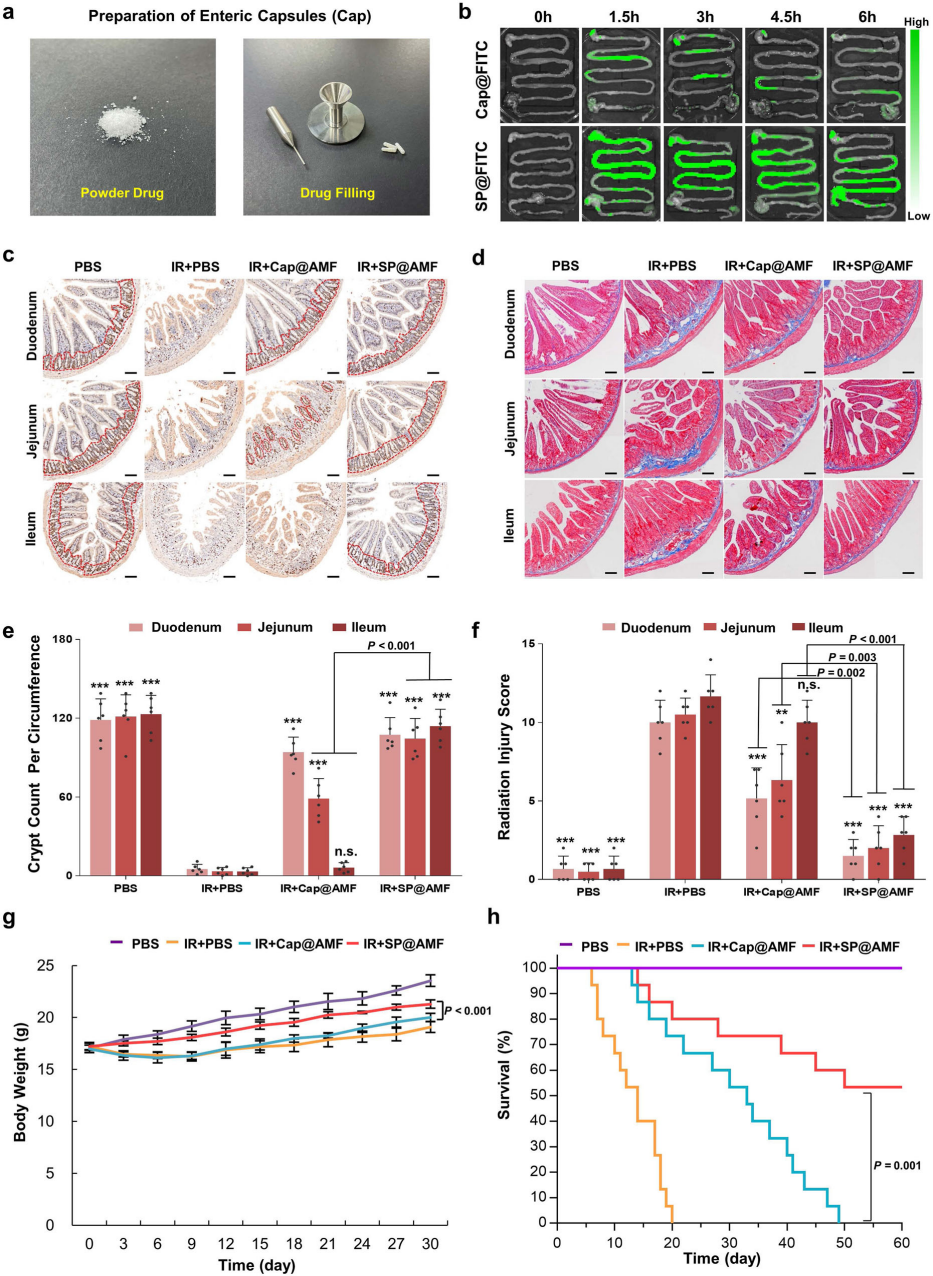
SP@AMF shows more comprehensive radioprotection on the whole small intestine compared with the enteric capsules of AMF (Cap@AMF). **a** The filling of drug powder into enteric capsules. **b** Fluorescence images of the mice’s gastrointestinal tract at 0, 1.5, 3, 4.5, and 6 h after the oral administration of Cap@FITC or SP@FITC (with equal amount of FITC). **c**–**f** Represented images (**c**) and the quantification (**e**) of the regenerating crypts (indicated by red dotted lines) in the small intestine (duodenum, jejunum, and ileum) at day 3 after being treated by sham irradiation + PBS (PBS group),12 Gy abdominal X-ray (IR) + PBS, IR + Cap@AMF, and IR + SP@AMF (*n* = 6 biologically independent animals). Represented images of the fibrosis formation (**d**) and the scoring of delayed radiation injury (**f**) at day 30 after treatments (*n* = 6 biologically independent animals). Scale bar = 100 µm. The data show means + SD. *P* was calculated using two-tailed *t*-test. *P versus IR + PBS group (*< 0.05, **<0.01, ***<0.001, n.s., no significance). Experiment was repeated three times independently with similar results. **g** Body weight of the mice (*n* = 6 biologically independent animals). The data show means ± SD. *P* was calculated using two-tailed *t*-test. **h** Survival curves of mice exposed to a fatal dose of abdominal IR (*n* = 15 biologically independent animals). Median survival: PBS, undefined (>60 days); IR + PBS, 14 d; IR + Cap@AMF, 33 d; IR + SP@AMF, undefined (>60 days). *P* was calculated using Log-rank (Mantel-Cox) test.

To further study the advantages of SP microcarrier in the oral drug delivery targeting the whole small intestine, a commonly-used commercial enteric-soluble capsules (Cap) (Fig. 6a) was used to encapsulate FITC and AMF to construct Cap@FITC and Cap@AMF, as comparisons to SP@FITC and SP@AMF respectively for the tests of distribution and efficacy. The fluorescence images of FITC in the post-harvest gastrointestinal tract showed that the fluorescence of Cap@FITC was discontinuous and only covered part of the small intestine (Fig. 6b). The quantification analysis (Supplementary Fig. 23) showed that compared with the SP@FITC group, the Cap@FITC group has a lower FITC concentration in all parts of the small intestine but a higher concentration in blood. This indicated that enteric-soluble capsules could increase the drug absorption into the bloodstream, as reported previously^28,29^, but not improve intestinal distribution. Subsequently, the indexes of early intestinal radiation injury (Fig. 6c–e and Supplementary Fig. 24) and delayed injury (Fig. 6d–f) were evaluated to compare the radioprotective effect of Cap@AMF and SP@AMF. As shown in Fig. 6c–f, the analysis on regenerating crypts (Fig. 6c–e) and fibrosis formation (Fig. 6d–f) showed that Cap@AMF had a mild protective effect on the proximal parts (duodenum and jejunum) and no protective effect on the distal parts (ileum) of the small intestine. In comparison, SP@AMF showed higher effective protection on all parts of the small intestine. Similar trends were observed in the evaluation of villus length (Supplementary Fig. 24a, b), DNA DSB (Supplementary Fig. 24c, d), and pro-inflammatory cytokines (Supplementary Fig. 24e–g). Meanwhile, the effects of SP@AMF on weight gain (Fig. 6g) and survival time (Fig. 6h) were better than those of Cap@AMF.

These results suggested that the use of the microalgae carrier, SP, significantly improved the radioprotective effect of AMF in the whole small intestine, which could not be achieved by the enteric-soluble capsules. This further indicated that except for protecting the drug from gastric degradation (which can also be solved by enteric-soluble capsulations), a prolonged and extensive intestinal biodistribution showed by SP is also crucial, especially for the radioprotection on distal parts of the small intestine.

### Effect on the radiotherapy of orthotopic colorectal cancer

**Fig. 7:**
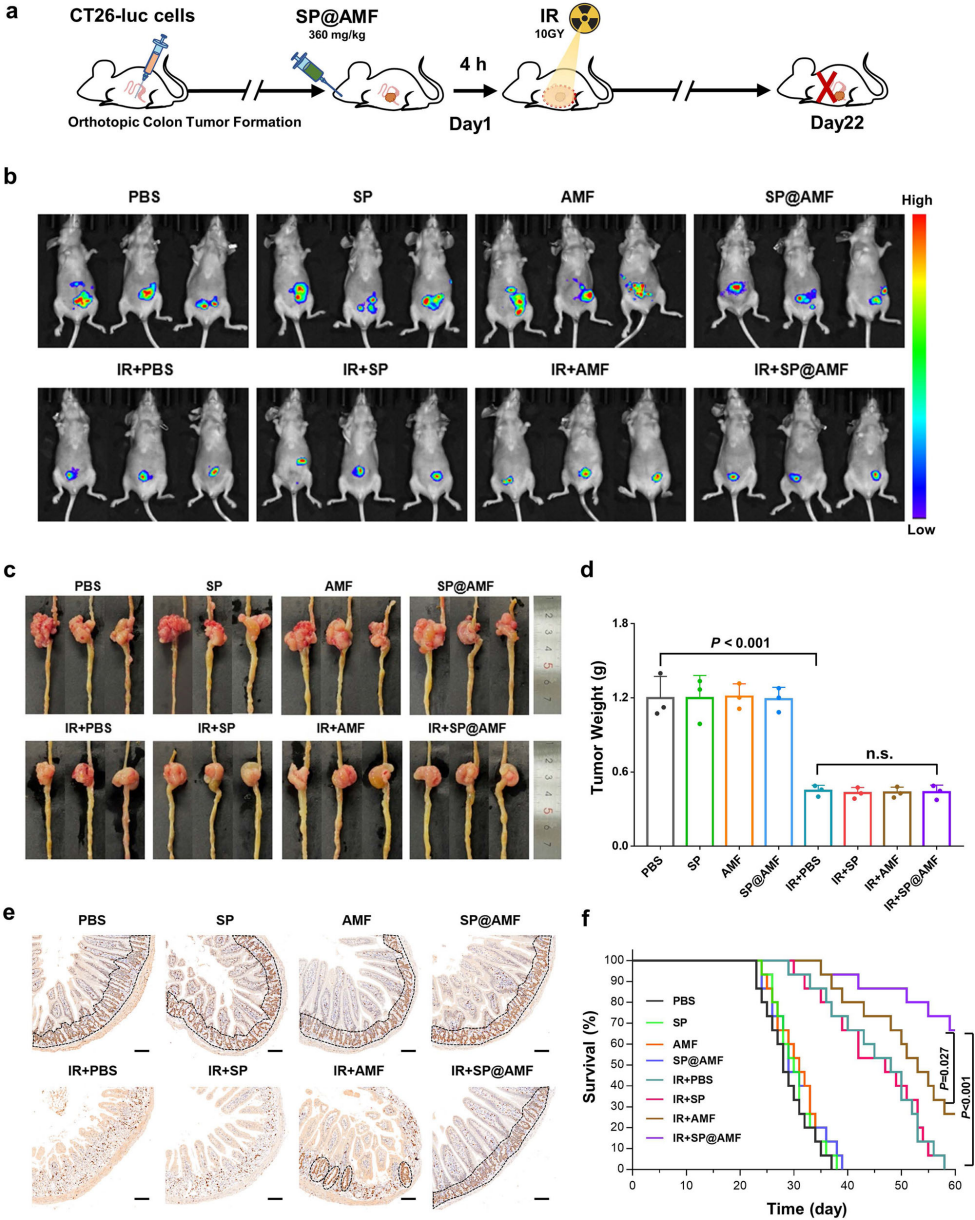
SP@AMF does not influence the regression of orthotopic colorectal tumor while protecting the normal intestine in radiotherapy. **a** Schematic illustration of the experiment protocol. **b**–**d** Fluorescence images (**b**), photographs (**c**), and weights (**d**) of the mice’s tumors at day 22 after being treated by sham irradiation + PBS (PBS group), 10 Gy abdominal X-ray (IR) + PBS, IR + Cap@AMF, and IR + SP@AMF (*n* = 3 biologically independent animals). The data show means + SD. *P* was calculated using two-tailed *t*-test. **e** Represented IHC images of the small intestine stained by Ki67. The black dotted line indicates the Ki67-stained regenerating crypts. Scale bar = 100 µm. Experiment was repeated three times independently with similar results. **f** Survival curves of mice (*n* = 15 biologically independent animals). Median survival: PBS, 28 days; SP, 30 days; AMF, 31 days; SP@AMF, 29 days; IR + PBS, 48 days; IR + SP, 47 days; IR + AMF, 53 days; IR + SP@AMF, undefined (>60 days). *P* was calculated using Log-rank (Mantel-Cox) test.

To evaluate the effect of SP@AMF on tumor radiotherapy, the tumor growth of the nude mice bearing orthotopic colorectal tumors was measured after the gavage of SP@AMF and abdominal X-ray irradiation (IR + SP@AMF group) (Fig. 7a). In addition to IR + PBS, IR + SP, and IR + AMF irradiation groups, PBS, SP, AMF, and SP@AMF feeding without irradiation groups were also used as comparisons. Both the in vivo fluorescence images (Fig. 7b) and the measurement of tumors (Fig. 7c, d) showed that the tumors in the four irradiated groups were significantly inhibited compared with the non-irradiated groups, indicating the important anti-cancer effects of radiotherapy. Notably, there was no significant difference in tumor weights between IR + SP@AMF and IR + PBS (Fig. 7d), demonstrating that SP@AMF did not protect tumors from radiation damage. Meanwhile, the small intestines in the IR + SP@AMF group were well protected from the radiation-induced injury while the small intestines in the other three irradiated groups were injured in different degrees (Fig. 7e). Furthermore, the usage of SP@AMF effectively improved the survival time of the irradiated mice (Fig. 7f). Importantly, the protective effect of SP@AMF was significantly superior to AMF (Fig. 7e, f), which confirmed the conclusions of the effect testing carried out in mice without tumors (Figs. 4–5). Collectively, these results demonstrated that SP@AMF can selectively protect the normal intestine and improve survival without affecting the radiotherapeutic effect against colorectal tumors.

### Protective effect on gut microbiota

**Fig. 8:**
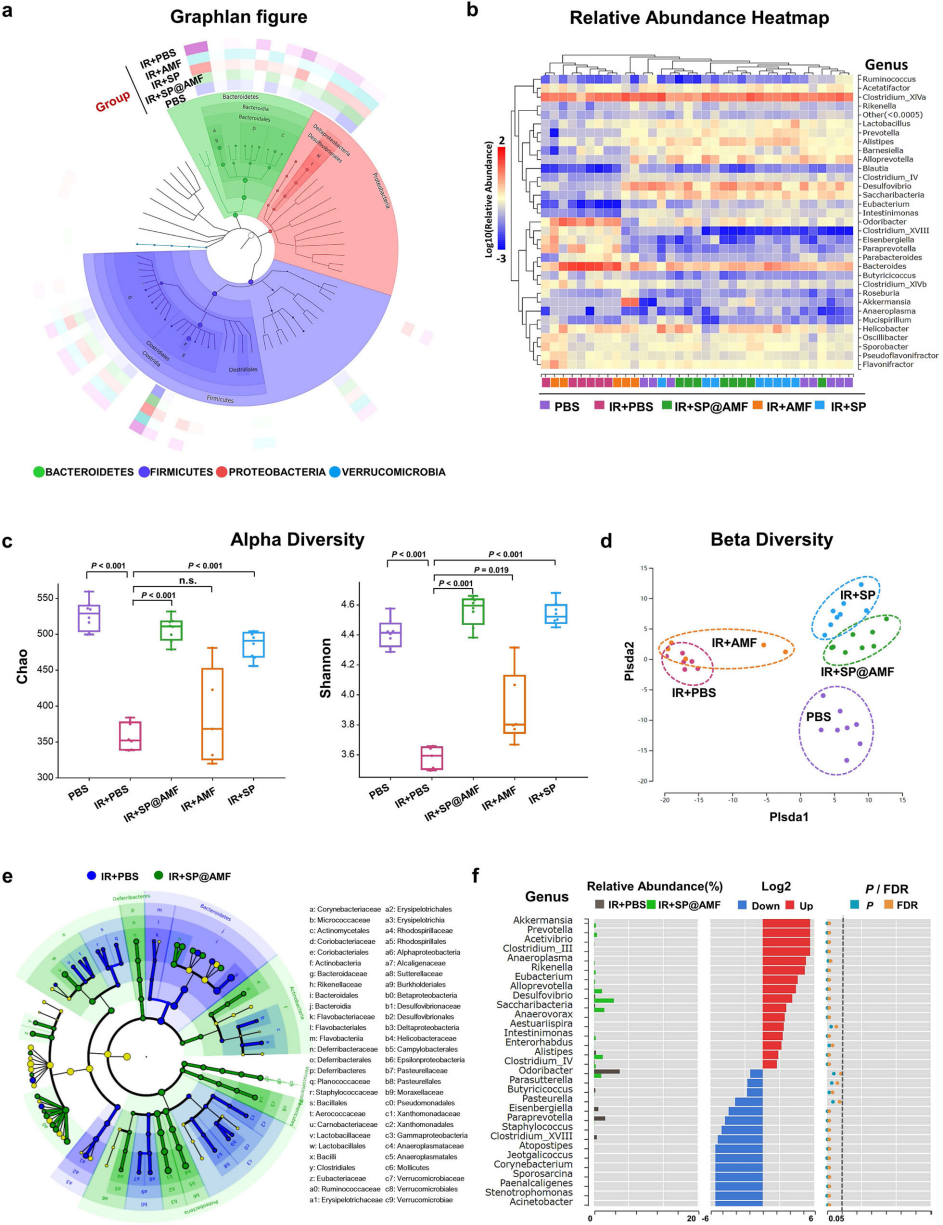
16S rRNA gene sequencing analysis shows the protective effect of SP@AMF on the gut microbiota of irradiated mice. **a** Graphlan figure, depicting the taxonomic association between the microbiome communities from different groups. A Bacteroidaceae; B Bacteroides; C Porphyromonadaceae; D Prevotellaceae; E Lachnospiraceae; F Clostridium XlVa; G Ruminococcaceae; H Desulfovibrionaceae; I Desulfovibrio. **b** Relative abundance heatmap of the gut microbiota of the samples in different groups in genus level. **c** Alpha diversity boxplot, in which the index Chao represents the community richness, and the index Shannon represents the community diversity. *n* = 8 for PBS, IR + SP, and IR + SP@AMF; *n* = 6 for IR + PBS; *n* = 5 for IR + AMF (representing biologically independent animals). Results are presented as the boxes’ bounds (the 25th to 75th percentile) and lines representing maxima, medians, and minima. *P* was calculated using two-tailed *t*-test. **d** Beta diversity PLS-DA (partial least-squares discrimination analysis) figure, visualizing the differences in the microbiota composition between groups through the distance in x-coordinate and y-coordinate. **e** LEfSe taxonomic cladogram, depicting the taxonomic association between the microbiome communities from IR + PBS and IR + SP@AMF. **f** The analysis of significant differences of the microbiota between IR + PBS and IR + SP@AMF, based on the Wilcoxon rank-sum test. *P* was calculated using two-tailed *t*-test.

We next studied the protective effect of SP@AMF on the gut microbiota of irradiation-treated mice by analyzing 16S rRNA gene sequencing of extracted fecal bacteria. The Graphlan figure (Fig. 8a) depicted the taxonomic association between the microbiome communities and generally showed the structure and relative abundance of gut microbiota in different treatment groups. The inner ring is the taxonomic cladogram. Each circle represented a level: phylum (in four different colors), class, order, family, and genus. The size of the nodes in the branching tree represents the abundance of the species. The outer ring is the abundance heat map, in which each ring with one color represents a group and the color depth varies with the abundance of species. It showed that irradiation (IR + PBS group) reduced the abundance (shown as color depth) of *Prevotellaceae* in *Bacteroidetes* and the *Desulfovibrio* in *Proteobacteria*, compared with normal gut microbiota (PBS group). By contrast, the species in the IR + SP@AMF group kept similar abundance with the PBS group, suggesting the protective effect of the SP@AMF on the irradiated gut microbiota. In the relative abundance heatmap of the gut microbiota (in genus level) of all samples from different groups (Fig. 8b), the color depth (red or blue) represents the relative abundance (log(*x* * 10^2^), *x* = relative abundance) of the species and the horizontal distance between two samples representing the similarity in species composition between them. Compared with the IR group, the IR + SP@AMF group and IR + SP group increased the relative abundance of several beneficial bacteria genus including Lactobacillus, Prevotella, Alistipes, and Alloprevotella, which were reported performing positive effects for intestinal inflammatory diseases^30–33^. And the genus composition of the samples in the IR + SP@AMF group and IR + SP group were more similar with the healthy mice.

According to the alpha diversity analysis (Fig. 8c and Supplementary Fig. 26), the microbiota community richness (Chao and Ace) and diversity (Shannon and Simpson) in the IR + PBS group were significantly lower than those in the healthy mice (PBS group). Compared with the IR + PBS group, the alpha diversity index of IR + SP@AMF and IR + SP groups was significantly improved, while no similar effect was observed in the IR + AMF group. Besides, the PLS-DA figure of beta diversity (Fig. 8d) revealed that, compared with other groups, the microbiota composition in the IR + SP@AMF group was the nearest to that in the PBS group, and that in the IR + SP group was close to that in the IR + SP@AMF group. Furthermore, the LEfSe (Fig. 8e) and significant difference analysis (Fig. 8f) were employed to compare the differences in bacterial community between IR + SP@AMF and IR + PBS groups. Compared with the IR + PBS group, there were more beneficial bacteria in the IR + SP@AMF group such as *Lactobacillaceae*, and *Helicobacteraceae* (Fig. 8e), which decreased the intestinal permeability and enhanced the intestinal barrier by producing short-chain fatty acids (SCFA)^34^, while the genus *Saccharibacteria* and *Clostridium IV* (Fig. 8f) were associated with resistance to the external invasion^35,36^.

### Long-term safety profiles

Finally, the long-term safety characteristics of SP@AMF were studied. After a month of daily gavage of different formulations, blood and major organs of the treated mice were harvested for further analysis. The results indicated that the long-term administration of SP and SP@AMF had no adverse effects on the main indexes, whereas the administration of AMF had obvious adverse effects on two hematological factors (WBC and MCH) and two serum biochemical factors (AST and CREA) (Fig. 8b). Although there were no obvious pathological changes (Fig. 8c), weight gain was slowest in the mice after AMF treatment (Fig. 8d), and death of mice even began 20 days after irradiation (Fig. 8e). The results demonstrated that the usage of microalgae carriers effectively avoided the long-term toxicity of oral administrated AMF.

## Discussion

As a helical microalga composed of multiple cells, SP has natural aqueous channels and junctional pores on the cell wall for the substances’ transmembrane exchange and slime secretion^37,38^. These natural channels allow small molecules to diffuse into the SP cell under the force of osmotic pressure^18,39^. In this study, SP was first lyophilized (dehydration) followed by adding to AMF solution (rehydration) (Fig. 1a, b), in which the dehydrated SP would undergo an extra flow-mediated drug loading during the rehydration process^18^ thus resulting in higher drug loading capacity (Supplementary Fig. 1). Importantly, the mature large-scale production of the microalga material and the facile drug loading strategy allow the mass preparation of SP@AMF (Fig. 1c), which provide significant potential for the future translation of SP@AMF. Owing to the instability of AMF in gastric acid^8^, its adequate distribution in the intestinal environment is an important concern. In the releasing examination we carried out, even pre-treated by SGF, SP could keep structure (Supplementary Fig. 5) and slowly release AMF into SIF (Fig. 1e). This may be attributed to the intrinsic characteristics of SP. Firstly, SP has a cell wall containing multilayers of glucan and peptidoglycan polymers^40^ which can provide physical protection against the structural destruction from the harsh gastric environment. Secondly, SP contains abundant alkaline minerals^41^, such as Na, K, Ca, and Mg, which can mildly neutralize the gastric acid to offer a relatively drug-friendly environment (Fig. 1f).

As mentioned, the injured parts of the small intestine tend to be different between individual patients^3^. So, another challenge in intestinal radioprotection of AMF is how to achieve comprehensive protection of the entire small intestine, especially the distal parts (ileum), because the soluble small-molecule drugs are easily washed away by intestinal fluid and absorbed into circulation in the proximal small intestine^10,11^. As a microcarrier with 200–500 μm of size, SP showed preferential retention between the intestinal villi (300–600 μm) (Fig. 3c–e) and progressive degradation in the intestinal tract (Fig. 3f). These characteristics enabled SP to provide a comprehensive and significant drug distribution throughout the small intestine, including the ileum (Fig. 3a, b, g–j). As a result, SP@AMF not only prolonged the radioprotective effect of AMF in vitro (Fig. 2, and Supplementary Figs. 7–10) but also improved the in vivo radioprotection of the whole small intestine, especially the ileum (Figs. 4–5 and Supplementary Figs. 20 and 21). Even compared with the enteric-soluble capsules of AMF(Cap@AMF) which can also protect the drug from gastric destruction, SP@AMF still showed a better protective effect on the distal small intestine (Fig. 6c–f and Supplementary Fig. 24). This might be explained by the capsules’ lower and discontinuous drug distribution in the intestine (Fig. 6b and Supplementary Fig. 23). These results suggested that SP is more applicable to drug delivery targeting the whole small intestine. However, more investigations are still needed in this area to clarify the strengths and weaknesses of SP versus other potential delivery systems.

Although many potential radioprotectants are being studied, their selective protective effect on normal tissues (and the related mechanisms) has rarely been validly proved^4^. As the only cytoprotective adjuvant approved by the FDA^4,5^, AMF has selective protection on normal tissues, but not on tumors. The widely recognized mechanisms of this are that normal tissues have higher vascular permeability and ALP (which could transfer AMF into the active form WR-1065) activity than tumor tissues^7^. Owing to this, SP@AMF also showed a selective radioprotection on the normal intestine cells or tissues without reducing the radiotherapy efficacy of tumor cells or tissues (Figs. 2c–f and 7b–e). Furthermore, SP@AMF effectively improved the survival time of the tumor-bearing mice after the radiotherapy (Fig. 7f). These findings have important implications for the transformation and application of SP@AMF in the clinical radiotherapy of abdominal/pelvic cancer.

**Fig. 9:**
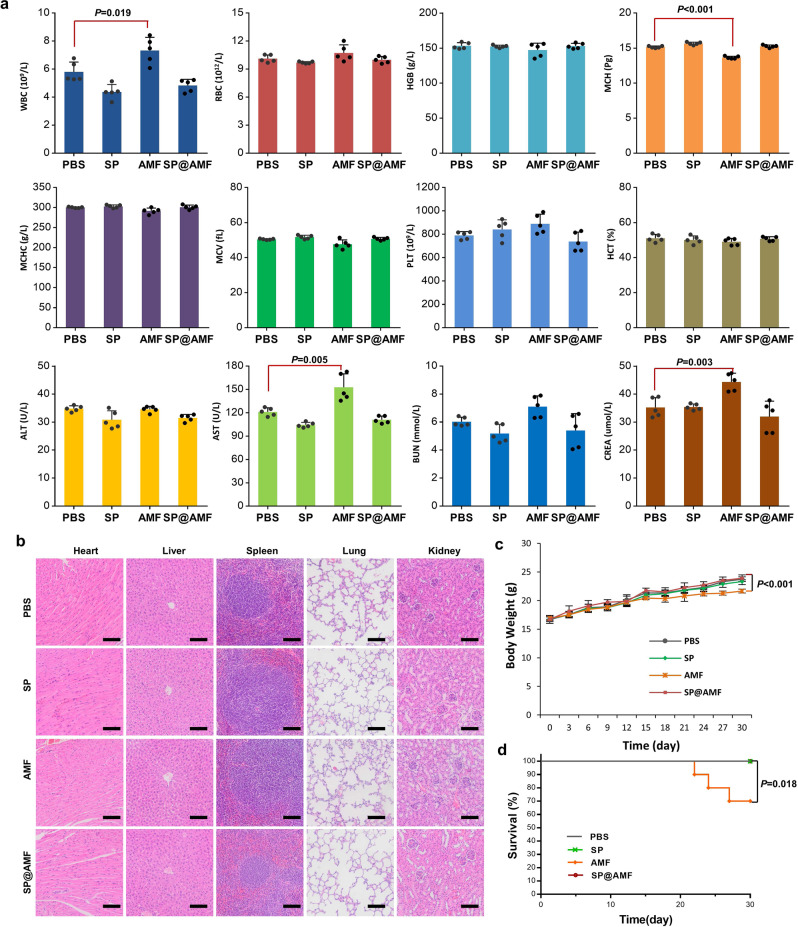
SP@AMF shows higher security in long-term use compared with AMF. **a** Hematological tests and serum biochemicals tests of the mice after the daily administration of PBS, SP, AMF, or SP@AMF for 30 days (*n* = 5 biologically independent animals). The data show means + SD. *P* was calculated using two-tailed *t*-test. WBC white blood cells, RBC red blood cells, HGB hemoglobin, MCH mean corpuscular hemoglobin, MCHC mean corpuscular hemoglobin concentration, MCV mean cell volume, PLT blood platelet, HCT hematocrit, ALT alanine transferase, AST aspartate transferase, BUN blood urea nitrogen, CREA creatinine. **b** Represented HE images of the major organs of the mice after different treatments for 30 days. Scale bar = 200 μm. Experiment was repeated three times independently with similar results. **c** Body weight of mice (*n* = 5 biologically independent animals). The data show means ± SD. *P* was calculated using two-tailed *t*-test. **d** survival curves of mice (*n* = 10 biologically independent animals). Median survival: PBS, undefined (>30 days); SP, undefined (> 30 days); AMF, undefined (>30 days); SP@AMF, undefined (>30 days). *P* was calculated using Log-rank (Mantel-Cox) test.

Interestingly, the 16S rRNA gene-sequencing analysis of gut microbiota in Fig. 8 showed that SP@AMF was beneficial to maintain the health of the gut microbiota of irradiated mice, which seemed to be attributed to the function of SP. Notably, oral administration of SP has already been demonstrated to be conducive to the health of gut microbiota, as it is enriched with some prebiotics, such as cellulose, polyunsaturated fatty acids, etc.^42,43^. Previous studies have reported that radiation-induced dysbiosis can promote intestinal injury^44,45^, and healthy gut microbiota is essential for nutrient absorption and immunity^46–50^. Therefore, the benefits of SP on microbiome balance may be complementary in aiding intestinal radiological protection in cancer patients. However, some moderate changes of the microbiota composition in our analysis seemed inexplicable and the definite function of many gut bacteria in the radiation-induced intestinal injury was still unclear. Therefore, in-depth examinations that combine metagenomics, metabolomics, and transcriptomics are required in future studies. In addition, long-term safety studies suggested that the toxicity of AMF could be avoided by SP@AMF (Fig. 9), which would be critical for its use in clinical radiotherapy because one treatment course of commonly-used fractionated radiotherapy would last at least 4 weeks.

In summary, we successfully constructed SP@AMF, a natural microcarrier-based orally delivered system, to effectively prevent the healthy intestine from radiation-induced damage in radiotherapy. Benefiting from the comprehensive intestinal biodistribution, the natural microcarrier, SP, significantly improved the radioprotection of AMF on the whole intestine. Possessing significant superiority to a commercial capsulation, the benefits on flora balance, as well as the high safety for long-term use, SP@AMF shows great potential to be applied in clinical radiotherapy of abdominal/pelvic tumors. Our study used readily available natural material to facilely construct a competitive radioprotective strategy with high translational potential in cancer radiotherapy.

## Methods

### Synthesis and characterization of SP@AMF

The *S. platensis* (SP) (Guangyu Biological Technology, Shanghai, China) cultured in Zarrouk medium (Guangyu Biological Technology, Shanghai, China) was placed in a 30 °C illumination incubator (Bluepard, Shanghai, China) (Supplementary Fig. 1). SP was centrifuged (3200 × *g*, 10 min), washed thoroughly with phosphate buffered saline (PBS) to remove the medium, and was finally lyophilized into powder by a lyophilizer (Labconco, USA). The concentration of Amifostine (AMF) (Yuanye Bio-Technology, Shanghai, China) in PBS solution was calculated by measuring the absorbance at 204 nm with the corresponding standard curve by a UV-2600 spectrophotometer (Shimadzu, Japan) (Supplementary Fig. 3). SP (0.5 mg) was dispersed in a 10 mL of PBS solution that contains 5 mg of AMF. Then, the solution was softly shaken in a dark environment at 4 °C for 12 h. Afterward, SP@AMF was separated from the solution by centrifugation (3200 × *g*, 10 min). The pellet was then collected, washed, and lyophilized into powder. Finally, the power was weighed and stored in a sealed bottle in a dry and dark environment at 4 °C for later use. The successful synthesis of SP@AMF was characterized with the Fourier transform infrared (FTIR) spectra by FTIR spectra Nicolet NEXUS 470 (Thermo Fisher Scientific, USA). Optical microscope (Zeiss, Germany) and field emission scanning electron (SEM) microscopy (Hitachi SU-70, Japan) were used to observe the morphology of synthesized SP@AMF.

To measure the drug loading efficiency (DLE) of AMF using the different drug loading concentrations, 0.4, 0.8, 1.6, 3.2, and 4.8 mg of AMF was added into 10 mL of PBS containing 0.5 mg of SP. After 12 h of drug loading, the AMF concentrations in the supernatant were measured by UV–Vis spectroscopy. The DLE of AMF after different loading times (1, 3, 6, 12, and 24 h) were tested by the same method. DLE was calculated by dividing the entrapped AMF by the amount of SP@AMF. To characterize the drug-releasing profiles of SP@AMF in the intestinal tract after passing through the gastric environment, we firstly treated SP@AMF by SGF and then transferred it into SIF to test the releasing data of AMF. In detail, 1 mg SP@AMF (containing 0.57 mg of AMF) was dispersed into 1 mL of SGF, then was softly shaken at 37 °C for 1 and 2 h, respectively. The untreated SP@AMF was used as a control. Then, the solution was centrifuged (3200 × *g*, 10 min) and transferred the sedimentary SP@AMF into 5 mL of SIF. Afterward, the solution was softly shaken at 37 °C for 0, 0.5, 1.5, 3, 6, and 12 h. Finally, the supernatants were collected and analyzed to quantify the released amount of AMF. To test the capacity to neutralize gastric acid, 60, 100, 200, and 300 mg of SP@AMF were suspended into 10 mL of 37 °C SGF. After 10 min, the solutions were centrifuged (3200 × *g*, 10 min) and the pH value of the supernatant was measured.

### In vitro toxicity and radioprotective effect

Rat’s small intestinal epithelium cells, IEC-6 (ATCC CRL-1592, EK-Bioscience, Shanghai), were cultured in DMEM (Dulbecco’s Modified Eagle Medium) supplemented with 10% FBS, 1% antibiotics (100 U/mL penicillin and 100 μg/mL streptomycin), and insulin (0.1 U/mL) at 37 °C with 5% CO_2_ atmosphere. To test the toxicity of materials, IEC-6 cells were seeded in 96-well plates (1 × 10^4^ per well) overnight and then incubated with DMEM containing different concentrations of SP@AMF (12, 23, 47, 94, 188, 375, and 750 μg/mL SP, corresponding to 16, 31, 63, 125, 250, 500, and 1000 μg/mL AMF) for 24 h. The same amount of SP and AMF were added in the cells of the other two groups. Cell viabilities were measured by standard methyl thiazolyl tetrazolium (MTT) assay kit (YEASEN, Shanghai, China).

To evaluate the protective effect, IEC-6 cells were seeded in 96-well plates (1 × 10^4^ per well) overnight and incubated with DMEM containing PBS, SP (23 μg/mL), AMF (30 μg/mL), and SP@AMF (23 μg/mL SP, 30 μg/mL AMF), respectively. After incubation for 1 h, the plates were centrifuged (500 × *g*, 3 min) and the supernatant was softly removed. Then, fresh medium was added to the plates. After incubation for another 1, 2, 3, or 4 h, the plates were thoroughly washed to remove excess SP, AMF, or SP@AMF followed by being exposed to 6 Gy of X-ray at a dose rate of 8.415 Gy/min (X-RAD 160, PXi, USA). PBS group was sham-irradiated. After 12 h of culture, the cells were stained by Calcein-AM/PI double stain kit (YEASEN, Shanghai, China) and observed by a fluorescence microscope. The cell viability was analyzed accordingly. For colony formation assay, the irradiated cells were seeded into six well plates and cultured for 5 days. Cells were then fixed with methanol and stained with crystal violet staining solution (0.1%) for visualization of colonies. Surviving colonies with more than 50 cells were counted. Surviving fraction was calculated by dividing the colony counts by the seeded cell number. To assess the production of reactive oxygen species (ROS) of irradiated cells, ROS assay kit (YEASEN, Shanghai, China) was used to test ROS production. For the evaluation of DNA double-strand breaks (DSB), the irradiated cells were fixed by paraformaldehyde (4%, 30 min), treated with Triton X-100 (10 min) and BSA (5%, 1 h), then incubated with γH2AX antibody (Rabbit polyclonal IgG, ab11174, Abcam, Shanghai, China) (1:200 dilution) (4 °C, 12 h) and secondary antibody (Goat Anti-Rabbit IgG, ab6721, Abcam, Shanghai, China) (1:1000 dilution) (1 h). Nuclei of the cells were stained by 4-6-diamidino-2-phenylindole (DAPI). The stained cells were visualized by Laser Scanning Confocal Microscopy (Nikon A1, Japan). To evaluate if SP@AMF has a selective protective effect on normal intestinal cells under various X-ray doses, both IEC-6 cells and CT26 cells (ATCC# CRL-2638, EK-Bioscience, Shanghai)(mouse colorectal cancer cells) were cultured and treated with different materials as mentioned. Then they were incubated with the renewed medium for 4 h before being exposed to 0, 2, 4, and 6 Gy of X-ray, respectively. The irradiated cells were seeded into six well plates and cultured for 5 days for colony formation assay.

### In vivo biodistribution

All animal procedures in this study were performed according to the National Institute of Health Guide for the Care and Use of Laboratory Animals. All animal studies were approved by the Institutional Animal Care and Use Committee of Zhejiang University (AIRB-2021-952).

To visualize the biodistribution of the material, a small molecule fluorescent probe, FITC, was loaded into SP to construct SP@FITC. The synthetic of SP@FITC: SP (0.5 mg) was dispersed in 10 mL of PBS solution that contains 1 mg of FITC. Then, the solution was softly shaken in a dark environment at 4 °C for 12 h. Afterward, SP@FITC was separated from the solution by centrifugation (3200 × *g*, 10 min). The sediment was then collected and thoroughly washed for further usage. Six-week-old female Balb/c nude mice were fasted for 12 h, and then given SP@FITC PBS suspension or FITC PBS solution by oral gavage at the dose of 15 mg FTTC/kg. After 0, 1.5, 3, 4.5, and 6 h, the mice were imaged by an IVIS Lumina LT Series III (Perkin Elmer, USA) using the channel of FITC (excitation wavelength: 445–490 nm, emission wavelength: 515–575 nm). The fluorescence intensity in the abdominal area (up to the diaphragm, down to the pelvis) was analyzed by Living Image 4.5 software (Perkin Elmer, USA). The results were shown by calculating the ratios to the untreated group.

For the quantitative analysis of FITC distribution in intestinal tissue and blood, 6-week-old female Balb/c nude mice were treated following the same protocols as above. At the time points mentioned above, the mice’s gastrointestinal tract was collected and taken fluorescence images in the channel of FITC. Then the gastrointestinal content, small intestinal tissue (duodenum, jejunum, and ileum), and blood were collected. Small intestinal tissue was thoroughly washed, weighed, and processed into homogenate. The blood samples were rested at room temperature for 4 h, transferred to the 4 °C fridge, and stored overnight. The intestinal homogenate and blood samples were centrifuged for 10 min at the speed of 4500 × *g*. Then, the supernatant was collected and the absorbance of FITC in the supernatant was measured by MD-M5 enzyme marking instrument (Molecular Devices, USA) (excitation wavelength: 485 nm, emission wavelength: 520 nm). The concentration of FITC was analyzed according to the standard curves (Supplementary Fig. 13). At 3–4 h after gavage, the content of the stomach, small and large intestines were washed and processed and then observed by the optical microscope and SEM. To investigate the morphology and distribution of SP@FITC in the intestinal tract, the small intestines collected at 3–4 h after oral gavage was softly washed for once to remove the digestive fluid. Some of the tissue sections were fixed for DAPI staining and observation by a fluorescence microscope, and the other tissues were prepared for SEM observation.

For the pharmacokinetic study of AMF and its active metabolite, WR-1065, LC-MS analysis was used to detect their distribution in intestinal tissue and blood at 1, 2, 4, and 6 h after AMF or SP@AMF treatment. Six-week-old female Balb/c mice were fasted for 12 h, and then given SP@AMF PBS suspension or AMF PBS solution by oral gavage at the dose of 200 mg AMF/kg. After 0, 2, 4, and 6 h, the mice’s small intestine (duodenum, jejunum, and ileum) and blood were collected. Small intestinal tissue was thoroughly washed, weighed, and processed into homogenate. The intestinal homogenate and blood samples were centrifuged for 10 min at the speed of 4500 × *g*. Hundred and fifty microliter acetonitrile (ACN) was added into 50 μL supernatant of the intestinal homogenate or the blood serum for protein precipitation. After vortex for 1 min, the samples were centrifuged for 10 min at the speed of 4500 × *g*. The supernatant was collected and analyzed immediately or stored at −80 °C until analysis. A series of samples for calibration standards of AMF and WR-1065 (Amgicam, Wuhan, China) was prepared by diluting high-concentration stocks with the blank tissue homogenate and then prepared for analysis. The high-concentration stocks of AMF (Yuanye Bio-Technology, Shanghai, China) and WR-1065 were prepared in aqueous 50% acetonitrile solutions, respectively.

LC-MS instruments and conditions: The analysis was performed using Agilent 1290 series (Agilent, Waldbronn, Germany). Discovery@HS F5-3 column (10 cm × 2.1 mm, 3 μm particle size) was used for the separation of AMF and WR-1065. The column temperature was 35 °C. Gradient elution was applied with 0.1% formic acid (FA) and acetonitrile (ACN). The flow rate was 0.2 ml/min. The injection volume was 10 μL. The determination was performed by Agilent Technologies 6460 Triple Quad LC-MS system with Agilent Jet Stream Source electrospray ionization (ESI). The compounds were ionized in positive ion polarity mode. Ionization source conditions: capillary voltage +3000 V; gas temperature 325 °C, gas flow 5 L/min, nebulizer pressure 45 psi, sheath gas temperature 350 °C, sheath gas flow 11 L/min. Quantification was performed using multiple reaction (MRM) modes. Precursor ion →product ion, fragmentor voltage, and collision energy for AMF: 215 → 135, 80 V, 8 eV; for WR-1065: 135 → 90, 70 V, 12 eV. The LC-MS data was analyzed and output by Agilent Masshunter Workstation (Version B.07.00) software.

### Protective effect against early and delayed intestinal radiation injury

Six-week-old female Balb/c mice were randomly divided into five groups including PBS (normal control), IR + PBS (irradiation injury group), IR + SP, IR + AMF, and IR + SP@AMF. After 12 h of fasting, the mice in the IR + SP@AMF group were orally administered 360 mg/kg SP@AMF dispersed in 300 μL of PBS. For comparison, the mice were administered the same amount of PBS, SP, or AMF to the IR + SP@AMF group. Four h later, the abdomen (up to diaphragm, down to pelvis) of mice in IR groups were exposed to 12 Gy of X-ray at the dose rate of 8.415 Gy/min (X-RAD 160, PXi, USA). The time interval between SP@AMF treatment and radiation had been determined by testing the early intestinal injury of the mice that exposed to 12 Gy of X-ray at 1, 2, 4, and 6 h after the gavage of SP@AMF (360 mg/kg) 3 days after the X-ray irradiation (Supplementary Fig. 19).

Other body parts of mice were shielded by lead blocks. The mice in the PBS group were sham-irradiated. The mice were sacrificed 3 days after the irradiation to assess the early intestinal injury degree. The small intestine (duodenum, jejunum, and ileum) was collected and thoroughly flushed with PBS. Part of the intestinal tissues was fixed in 10% formalin for 24 h and processed for HE and immunohistochemistry (IHC) assays to investigate the expression of Ki67 and γH2AX. To avoid bias, the pathological sections were assessed by investigators who were blinded to the experimental treatments. The number of Ki67 staining regenerating crypts and γH2AX^+^ cells along the surrounding tissues of the intestinal cross-section were microscopically scored, and the length of small intestinal villi was measured by Image J (1.8.0_112, National Institutes of Health, USA). The rest part of the intestinal tissue was homogenated and analyzed by an enzyme-linked immunoabsorbent assay (ELISA) kit (Boster, Wuhan, China) to quantify the represented pro-inflammatory cytokines in radiation injury, including IL-1β, IL-6, and TNF-α. Other mice were divided and treated as mentioned above and were then weighed for one month. At the end of the month, the small intestine of mice was harvested for Masson Trichrome staining to observe the fibrosis and score the degree of delayed radiation injury according to the standards in the previous study^27^ (Table S1). To assess the effect on survival, mice were orally administered with PBS, SP, AMF, or SP@AMF followed by being given and a fatal dose of abdominal irradiation (16 Gy) except for the PBS group. This fatal dose was determined according to the results of the preliminary experiment: Six-week-old female Balb/c mice were respectively exposed to 12 and 16 Gy of X-ray abdominal radiation at the dose rate of 8.415 Gy/min. Then the mice were monitored to record survival time (Supplementary Fig. 22a).

### Biodistribution and radioprotective effect of the enteric capsules of AMF (Cap@AMF)

The enteric capsules (commercial capsulation with polyacrylate coating which dissolves in the intestine but not in the stomach) for mice, drug filling device, and oral delivery device were purchased from Yuyan Instruments (Shanghai, China). The capsule is mainly made up of gelatin and coated with polyacrylic resin material. It will dissolve in solutions with pH 6.3–6.8. The drug filling and delivery processes of capsules were carried out following the operation instructions (http://www.yuyanbio.com).

FITC powder was also weighted and filled into enteric capsules to construct Cap@FITC for the visualization of in vivo biodistribution. SP@FITC was prepared as described above. Six-week-old female Balb/c nude mice were fasted for 12 h and then given Cap@FITC or SP@FITC PBS suspension by oral gavage at the dose of 15 mg FTTC/kg. The collection of gastrointestinal tracts at different hours (0, 1.5, 3, 4.5, and 6) after the gavage, the fluorescence imaging, and the quantitative test of FITC in small intestinal tissue and blood were followed the same processes as described.

AMF powder was weighted and filled into enteric capsules to construct Cap@AMF for the evaluation of the radioprotective effect. Forty-eight 6-week-old female Balb/c mice were randomly divided into four groups including PBS (normal control), IR + PBS (irradiation injury group), IR + Cap@AMF, and IR + SP@AMF. The mice in the IR + SP@AMF group were orally administered 360 mg/kg SP@AMF dispersed in 300 μL of PBS. For comparison, the PBS and IR + Cap@AMF group was respectively given the same amount of PBS and AMF as that of the IR + SP@AMF group. Material and 12 Gy abdominal irradiation were given following the same protocols as above. Half of the mice were sacrificed 3 days after the irradiation to assess the early intestinal injury, while another half were monitored for 1 month to evaluate the body weight and the delayed intestinal radiation injury. Other mice were given the same treatment as above and a fatal dose of abdominal irradiation (16 Gy) except for the PBS group to monitor survival time for 60 days.

### Effect on the radiotherapy of orthotopic colorectal cancer

To establish an orthotopic colorectal cancer model, luciferase-expressing mouse colorectal cancer cells, CT26-luc (Sciencelight Biology, Shanghai), were first cultured to exponential growth stage. Then, the cells were suspended in PBS at the concentration of 10^7^ cells in 1 mL. Afterward, 6-week-old female Balb/c nude mice were anesthetized and sterilized, a 0.5 cm incision was made in the middle of its abdomen. The cecum was gently pulled out from the incision. Fifteen microliter of the cell suspension was slowly injected into the serous layer of the cecum. The injection point was softly pressed for a while, and the cecum was relocated. Finally, the abdominal wall was sutured layer by layer. When the length of the tumor reached about 3–5 mm determined by the fluorescence image, the mice were randomly assigned to one of the groups including PBS, SP, AMF, SP@AMF, IR + PBS, IR + SP, IR + AMF, and IR + SP@AMF. Materials were given following the same protocols as above. The X-ray dose was adjusted to 10 Gy according to the results of the preliminary experiment: When the length of the tumor reached about 3–5 mm determined by the fluorescence image, the nude mice were respectively exposed to 0 (as a comparison), 10, 12, and 16 Gy of X-ray abdominal radiation at the dose rate of 8.415 Gy/min. Then the mice were monitored to record survival time (Supplementary Fig. 22b). Bioluminescent images (BLI) of the mice were taken to monitor the tumors in vivo. Mice were intraperitoneally (i.p.) injected 150 mg/kg d-luciferin (Yeasen, Shanghai, China) and were taken BLI images by IVIS Lumina LT Series III (Perkin Elmer, USA) in 10 min. As a reference, different numbers of CT26-luc cells were seeded and incubated in 24-well plates to take BLI images (Supplementary Fig. 25). On day 3 after treatments, the small intestine was collected and sectioned for Ki67-staining to observe the radiation injury. On day 22 after treatments, the tumors on the large intestine were collected and weighted. Other tumor-bearing mice were treated, irradiated, and monitored for 60 days to record survival time.

### Effect on gut microbiota

To test the potential protective effect of SP@AMF on the gut microbiota, 6-week-old female Balb/c mice were randomly divided into five groups, including PBS, IR + PBS, IR + SP, IR + AMF, and IR + SP@AMF. After the material and X-ray irradiation was given as mentioned, the oral administration of different materials was continued every day for the following 7 days. Afterward, the feces samples were collected for the 16S rRNA gene sequencing (BGI Co., Ltd, Shenzhen, China). The protocols of DNA extraction and library construction were included in Supplementary Information. The samples with the successfully constructed libraries were analyzed to illustrate the effect of different treatments on the gut microbiota (two in IR + PBS and three in the IR + AMF group were failed in the database construction).

The microbial community DNA was extracted using MagPure Stool DNA KF kit B (Magen, China) following the manufacturer’s instructions. DNA was quantified with a Qubit Fluorometer by using a Qubit dsDNA BR Assay kit (Invitrogen, USA) and the quality was checked by running an aliquot on 1% agarose gel. Variable regions V4 of bacterial 16S rRNA gene was amplified with degenerate PCR primers, 515F (5-GTGCCAGCMGCCGCGGTAA-3) and 806R (5- GGACTACHVGGGTWTCTAAT-3). Both forward and reverse primers were tagged with Illumina adapter, pad, and linker sequences. PCR enrichment was performed in a 50 μL of reaction containing 30 ng of template, fusion PCR primer, and PCR master mix. PCR cycling conditions were as follows: 95 °C for 3 min, 30 cycles of 95 °C for 45 s, 56 °C for 45 s, 72 °C for 45 s, and final extension for 10 min at 72 °C for 10 min. The PCR products were purified using Agencourt AMPure XP beads and eluted in Elution buffer. Libraries were qualified by the Agilent Technologies 2100 bioanalyzer. The validated libraries were used for sequencing on Illumina HiSeq 2500 platform (BGI, Shenzhen, China) following the standard pipelines of Illumina, and generating 2250 bp paired-end reads.

### Long-term safety profiles

To assess the long-term safety of SP@AMF, 6-week-old female Balb/c mice were daily given 360 mg/kg SP@AMF for 30 days in a row. The mice in other groups were given the same amount of PBS, SP, or AMF as the SP@AMF group. The body weight of mice was recorded. The survival time was monitored. After a month, the mice were euthanized, and the blood and major organs were collected for the hematological and pathological examinations.

### Statistical analysis

All statistical analyses were performed by GraphPad Prism v.7.00 (GraphPad Software). The statistical significance was indicated as *P < 0.05; **P < 0.01; ***P < 0.001; n.s., no significance.

### Reporting summary

Further information on research design is available in the Nature Research Reporting Summary linked to this article.

## Supplementary information

Supplementary Information

Reporting Summary

## Data Availability

All relevant data supporting the key findings of this study are available within the article and its Supplementary Information files or from the corresponding author upon reasonable request. Source data are provided with this paper.

## Source data

Source Data
