# Supplementary material for: Microalgae-based oral microcarriers for gut microbiota homeostasis and intestinal protection in cancer radiotherapy
Source: Nat Commun. 2022 Mar 17;13:1413. doi: 10.1038/s41467-022-28744-4 (PMC8931093; doi:10.1038/s41467-022-28744-4)
Supplement: Supplementary file 1 — Supplementary Information [file 41467_2022_28744_MOESM1_ESM.pdf]

Supplementary Information for

Microalgae-based oral microcarriers for gut microbiota homeostasis and intestinal protection in cancer radiotherapy

Dongxiao Zhang[1,2]*, Danni Zhong[1,2]*, Jiang Ouyang,[3]* Jian He[2], Yuchen Qi[2], Wei Chen[3], Xingcai Zhang[5,6]†, Wei Tao[3]†, and Min Zhou[1,2,4]†

[1] Eye Center, The Second Affiliated Hospital, Zhejiang University School of Medicine, Hangzhou, 310029, China.

[2] Institute of Translational Medicine, Zhejiang University, Hangzhou, 310029, China

[3] Center for Nanomedicine and Department of Anesthesiology, Brigham and Women's Hospital, Harvard Medical School, Boston, 02115, MA, USA

[4] School of Engineering and Applied Sciences, Harvard University, Cambridge, MA, 02138, USA

[5] School of Engineering, Massachusetts Institute of Technology, Cambridge, MA, 02139, USA

[6] State Key Laboratory of Modern Optical Instrumentations, Zhejiang University, Hangzhou, 310058, China

[7] Cancer Center, Zhejiang University, Hangzhou, 310058, China

*These authors contributed equally to this work.

† Corresponding author. Email: zhoum@zju.edu.cn (M.Z.), wtao@bwh.harvard.edu (W.T.), xingcai@mit.edu (X.Z.)

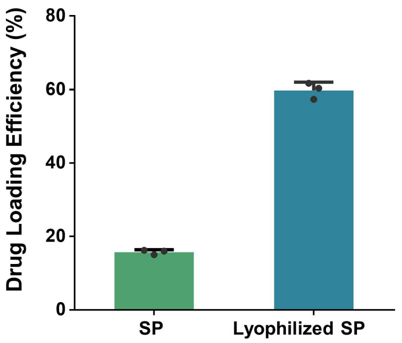

Supplementary Fig. 1. The drug loading efficiency of AMF in fresh SP and lyophilized SP (n = 3 biologically independent samples). Results are presented as means ± SD.

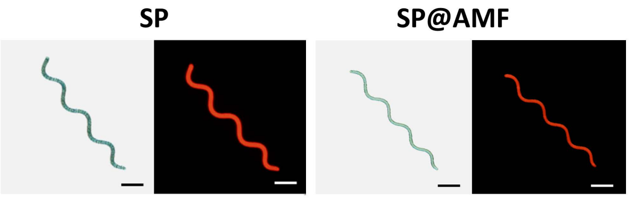

Supplementary Fig. 2. Bright-field images and fluorescence microscope images (red for SP's chlorophyll) of SP and SP@AMF, respectively. Scale bar = 20 μm. Experiment was repeated three times independently with similar results.

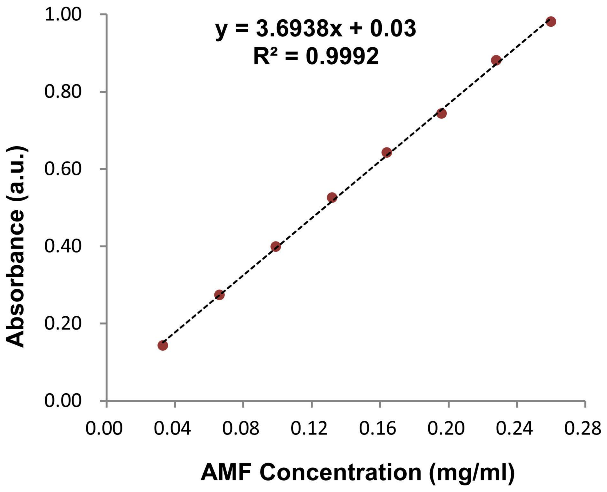

Supplementary Fig. 3. Standard curve of AMF in PBS solution calculated using the absorbance at 204 nm.

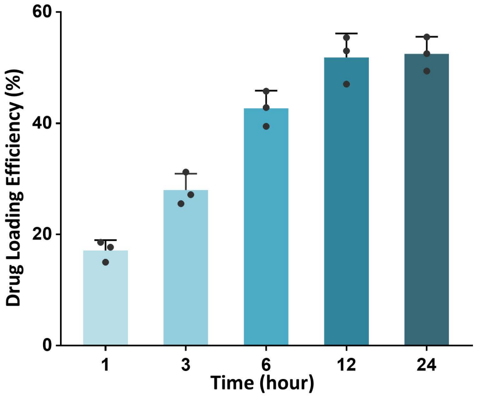

Supplementary Fig. 4. The drug loading efficiency of AMF in freeze-dried SP after 1, 3, 6, 12, and 24 h of drug loading (n = 3 biologically independent samples). Results are presented as means ± SD.

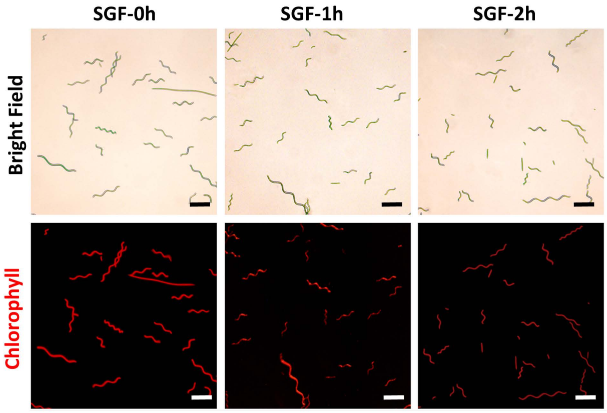

Supplementary Fig. 5. Bright-field and fluorescence microscope images (red for SP's chlorophyll) of SP@AMF treated by simulated gastric fluid (SGF) at 37°C for 0 (untreated), 1 and 2 h. Scale bar = 100 μm. Experiment was repeated three times independently with similar results.

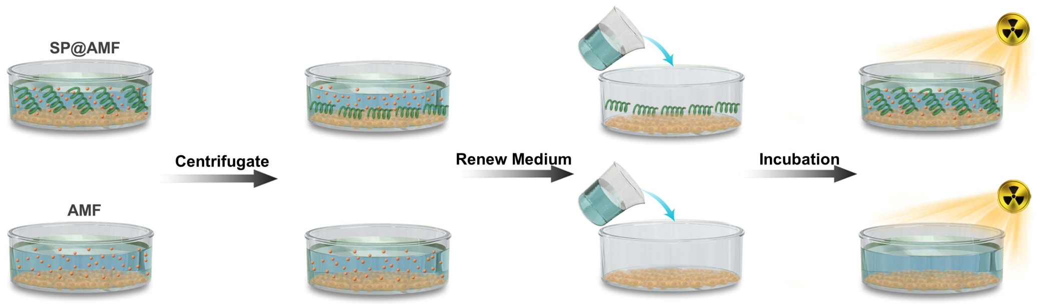

Supplementary Fig. 6. Schematic illustration of the cell experiments in Fig.2b and Supplementary Fig. 7-10.

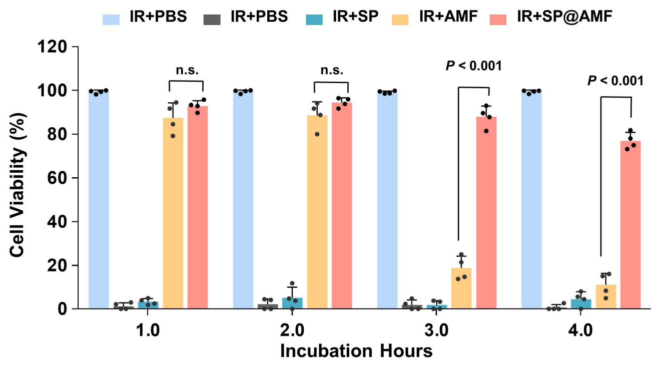

Supplementary Fig. 7. The viability of the IEC-6 cells irradiated by 6 Gy X-ray after 1, 2, 3, or 4 h of incubation with the renewed medium, determined by the Calcein-AM/PI fluorescence microscope images in Fig. 2b (n=4 biologically independent cells). The data show means + SD. *P* was calculated using two-tailed t test. n.s., no significance ($P > 0.05$).

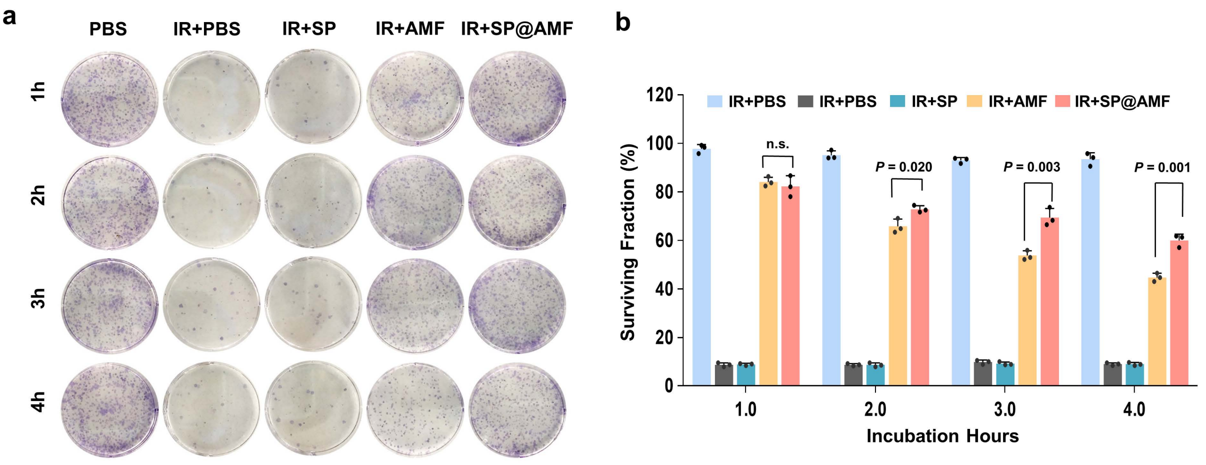

Supplementary Fig. 8. Crystal violet staining of the surviving colonies (a) and the quantification (b) of IEC-6 cells irradiated by 6 Gy X-ray after 1, 2, 3, or 4 h of incubation with the renewed medium (n=3 biologically independent cells). The data show means + SD. *P* was calculated using two-tailed t test. n.s., no significance ($P > 0.05$).

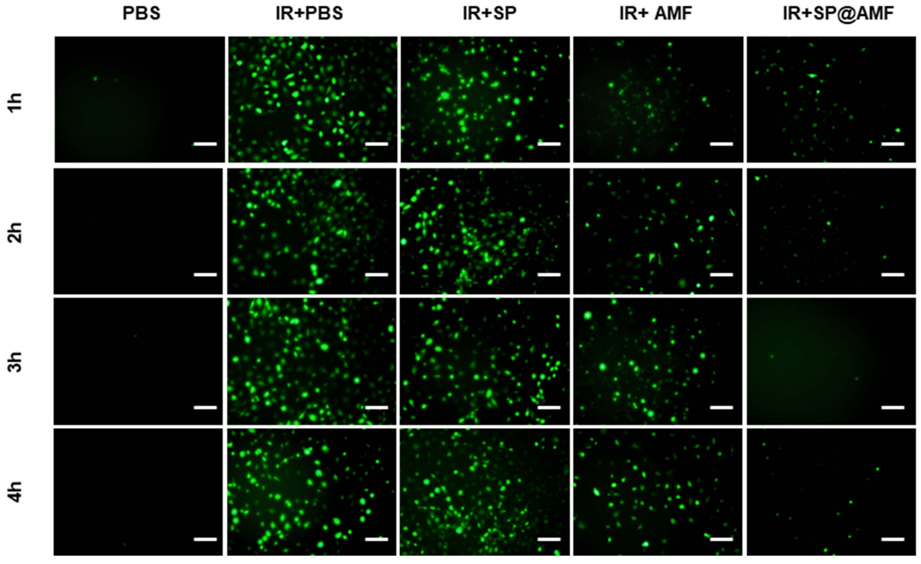

Supplementary Fig. 9. Fluorescence images of ROS production in IEC-6 cells after the exposure to 6 Gy X-ray irradiation (except for PBS group) after 1, 2, 3, or 4 h of incubation with the renewed medium in different groups, stained with ROS assay kit (green). Scale Bar =50 μm. Experiment was repeated three times independently with similar results.

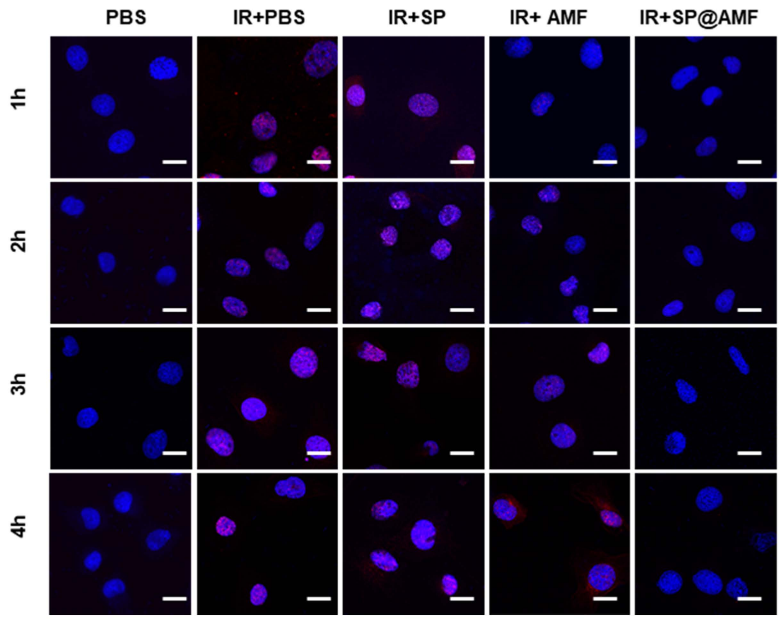

Supplementary Fig. 10. Immunofluorescence images showing the DNA double-strand break (DSB) of IEC-6 cells after the exposure to 6 Gy X-ray irradiation (except for PBS group) after 1, 2, 3, or 4 h of incubation with the renewed medium in different groups, in which γ H2AX was stained for DNA DSB (red) and DAPI was stained for nuclei (blue). Scale bar = 10 μm. Experiment was repeated three times independently with similar results.

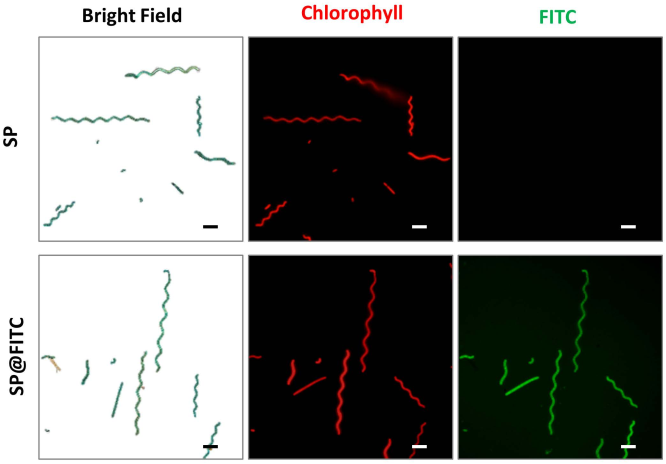

Supplementary Fig. 11. Bright field and fluorescence microscope (red for SP's chlorophyll, green for FITC) images of SP@FITC. Scale bar = 20 μm. Experiment was repeated three times independently with similar results.

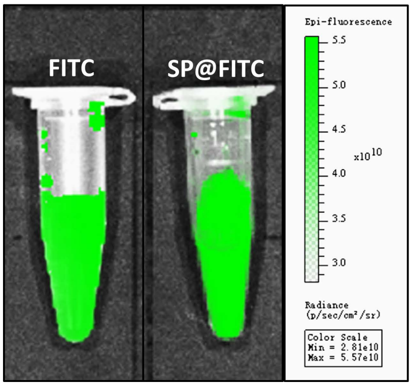

Supplementary Fig. 12. Fluorescence images of FITC and SP@FITC (with an equal concentration of FITC) that used for the observation of the in vivo biodistribution after oral administration. FITC channel: excitation wavelength = 445-490 nm, emission wavelength = 515-575 nm.

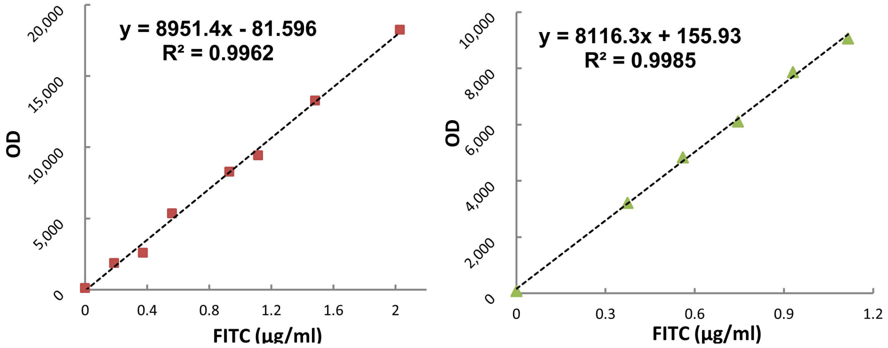

Supplementary Fig. 13. Standard curves of FITC in intestine homogenate (a) and blood serum (b) calculated using the absorbance at 515-575 nm (excitation spectrum: 445-490 nm).

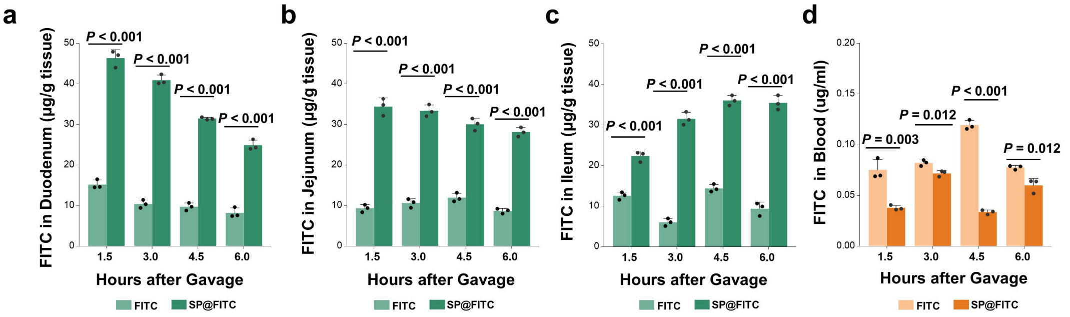

Supplementary Fig. 14. Quantification of FITC in the intestinal tissue of duodenum (**a**), jejunum (**b**), ileum (**c**), and the blood (**d**) at 0, 1.5, 3, 4.5, and 6 h after the oral administration of FITC or SP@FITC (with an equal amount of FITC) (n=3 biologically independent animals). The data show means + SD. *P* was calculated using two-tailed t test.

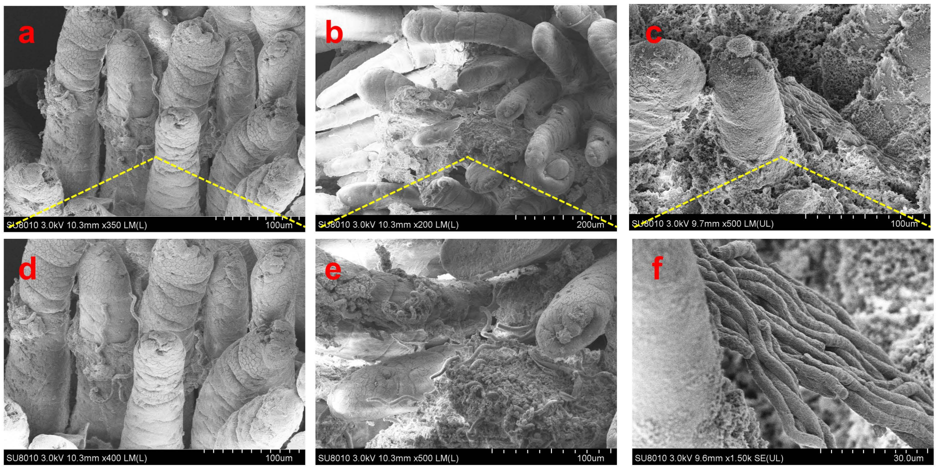

Supplementary Fig. 15. SEM images of SP@AMF on the surface of the ileum of mice. **a, d.** The original SEM images of Fig. 3c. Experiment was repeated three times independently with similar results.

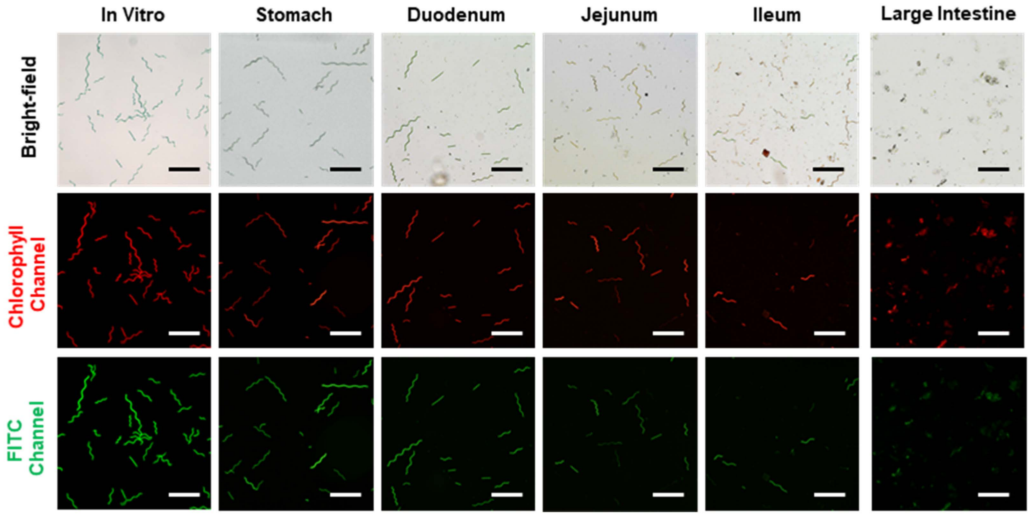

Supplementary Fig. 16. Bright field and fluorescence microscope (red, chlorophyll in SP; green, FITC) images of SP@FITC in the stomach, small and large intestines, at 4 h after the oral administration of SP@FITC. Scale bar = 200 μm. Experiment was repeated three times independently with similar results.

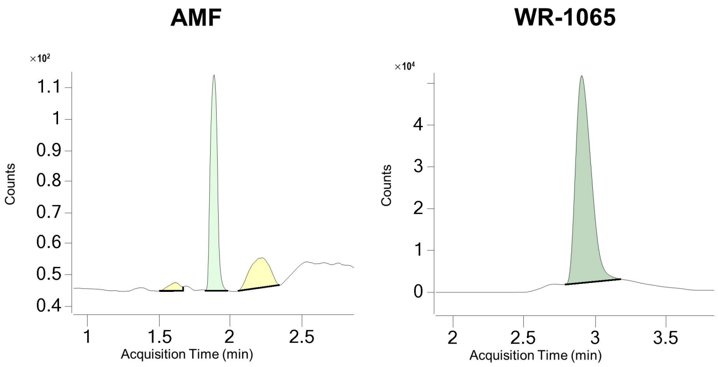

Supplementary Fig. 17. Representative LC-MS chromatograms of AMF (acquisition time 1.886 min) and its active metabolite WR-1065 (acquisition time 2.904 min).

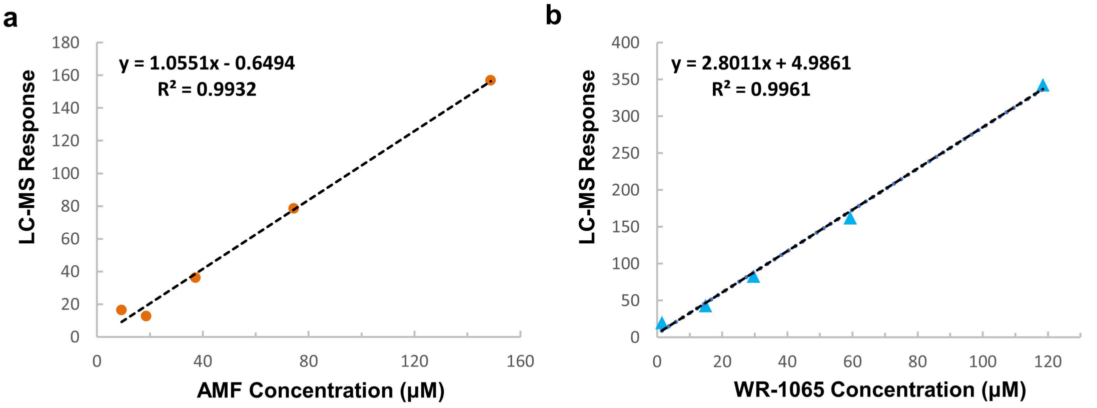

Supplementary Fig. 18. Standard calibration curves of AMF (**a**) and WR-1065 (**b**) detected by LC-MS.

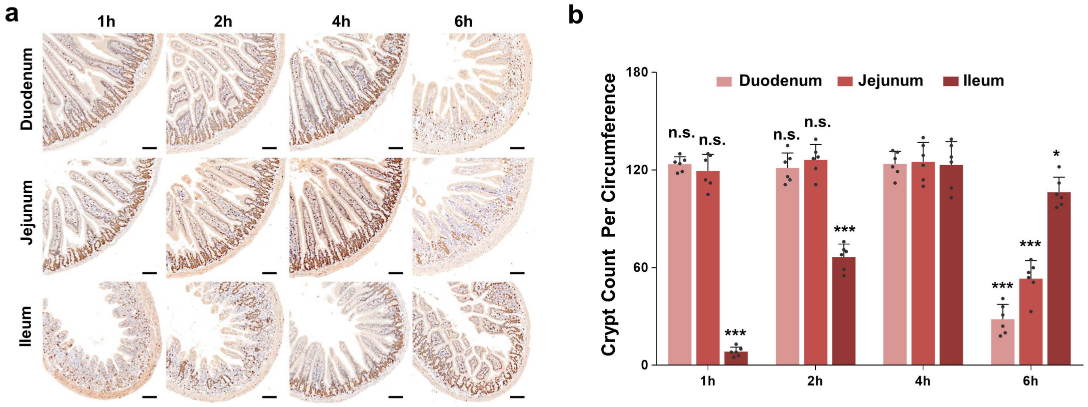

Supplementary Fig. 19. **SP@AMF performs optimal radioprotective effect on the regenerating crypts in all parts of the small intestine when used 4 h before radiation, compared with other time intervals (1, 2, and 6 h). a.** Represented Ki67 IHC images of the regenerating crypts in the small intestine (duodenum, jejunum, and ileum) of mice which have been irradiated by 12 Gy abdominal X-ray at 1, 2, 4, and 6 hours after the gavage of SP@AMF. Scale bar =100 μm. **b.** Quantification of the regenerating crypts in different groups (n=6 biologically independent animals). The data show means + SD. *P* versus 4 h group was calculated using two-tailed t test. **P*< 0.05, ***P* < 0.01, ****P* < 0.001, n.s., no significance. Experiment was repeated three times independently with similar results.

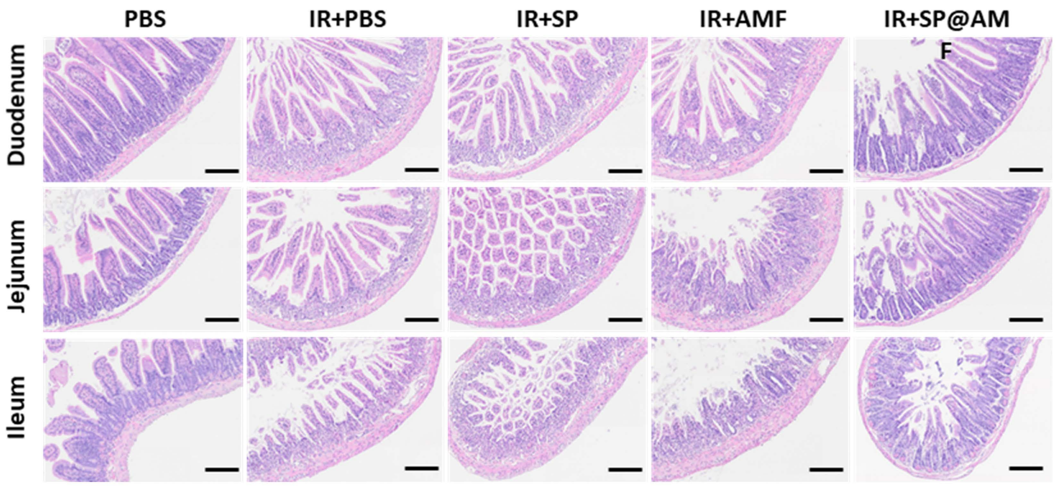

Supplementary Fig. 20. Representative images of HE-staining images of the small intestine of the mice treated by PBS + sham irradiation (PBS group), abdominal 12 Gy X-ray irradiation (IR) + PBS, SP, AMF, and SP@AMF. Scale bar = 200 μm. Experiment was repeated three times independently with similar results.

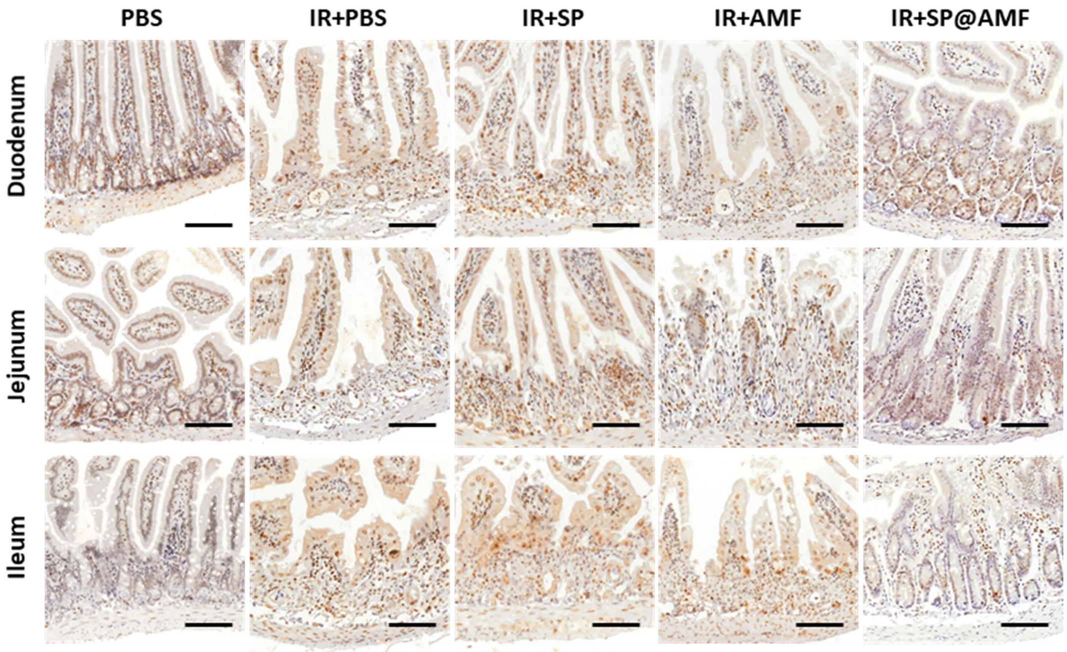

Supplementary Fig. 21. Representative IHC images of the γH2AX staining of the small intestine of the mice treated by PBS + sham irradiation (PBS group), abdominal 12 Gy X-ray irradiation (IR) + PBS, SP, AMF, and SP@AMF. Scale bar = 100 μm. Experiment was repeated three times independently with similar results.

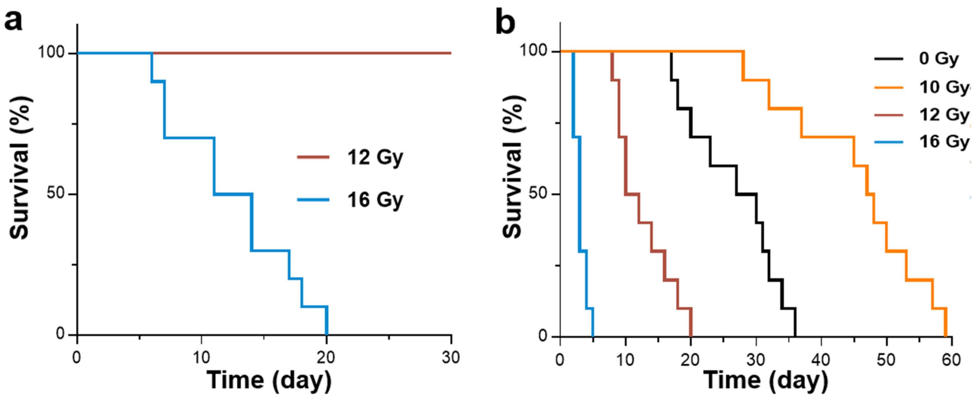

Supplementary Fig. 22. **a.** Survival curves of Balb/c mice exposed to various doses of X-ray abdominal irradiation (n=10 biologically independent animals). Median survival: 12 Gy, undefined (> 30 d); 16 Gy, 13d. *P* was calculated using Log-rank (Mantel-Cox) test. *P* < 0.001. **b.** Survival curves of Balb/c nude mice bearing orthotopic colorectal tumors exposed to various doses of X-ray abdominal irradiation (n=10 biologically independent animals). Median survival: 0 Gy, 28.5 d; 10 Gy, 47.5 d; 12 Gy, 11 d; 16 Gy, 3 d. *P* was calculated using Log-rank (Mantel-Cox) test. *P* < 0.001.

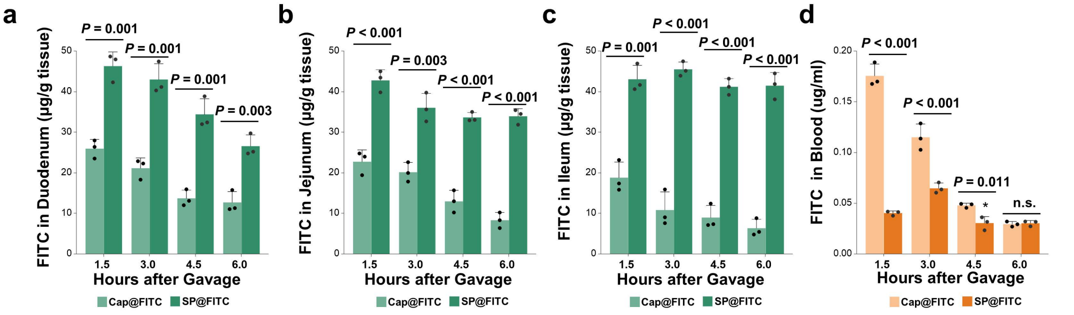

Supplementary Fig. 23. Quantification of FITC in small intestine tissue (duodenum, jejunum, and ileum) (a-c) and blood (d) of the mice at 0, 1.5, 3, 4.5, and 6 h after the oral administration of the enteric capsules of FITC (Cap@FITC) or SP@FITC (with equal amount of FITC) (n=3 biologically independent animals). The data show means + SD. *P* was calculated using two-tailed t test.

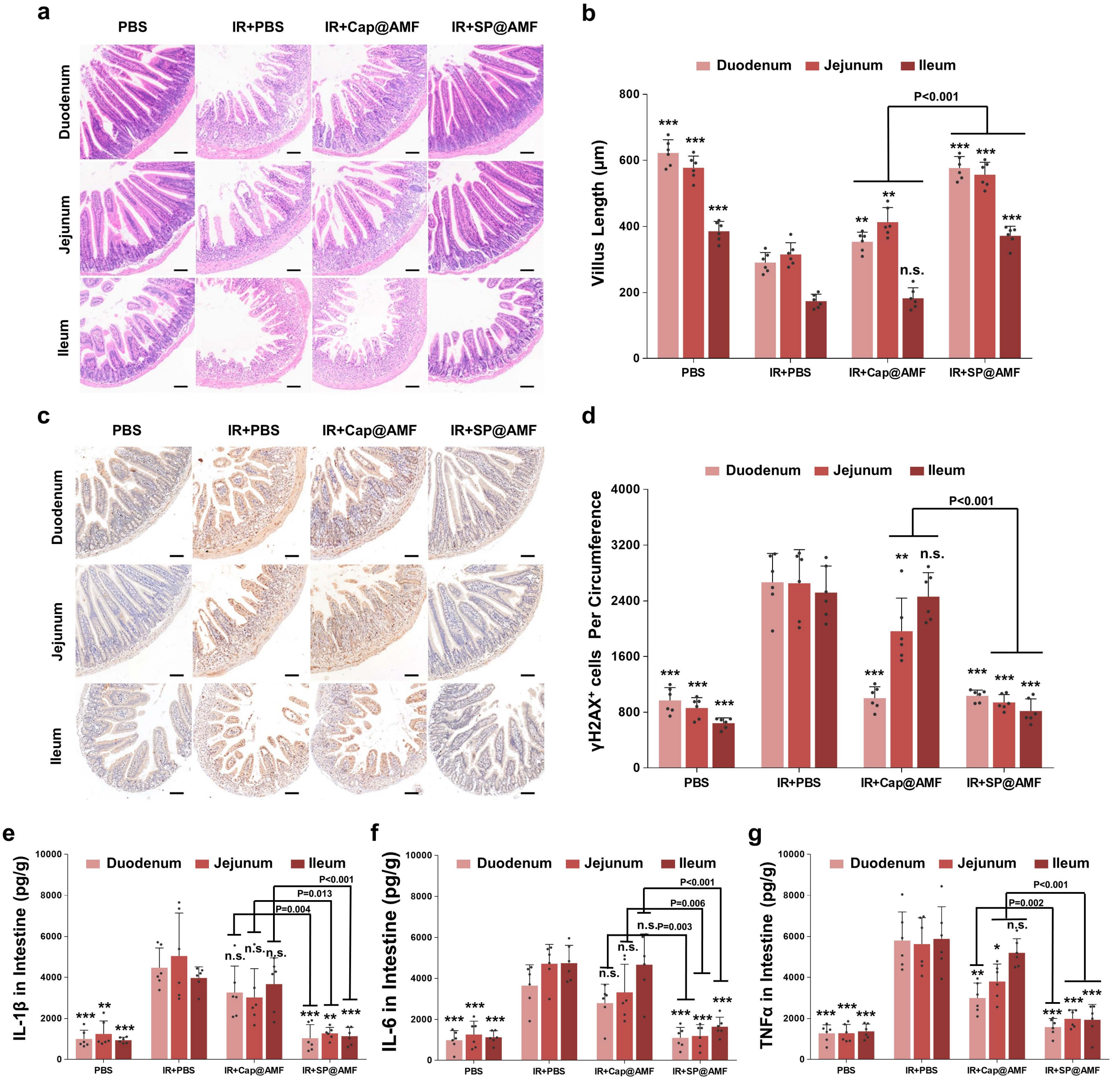

Supplementary Fig. 24. SP@AMF shows longer and more extensive radioprotection in the whole small intestine compared with the enteric capsules of AMF (Cap@AMF). a-d. Represented images and the quantification of the length of intestinal villi (**a, b**) and the γH2AX-positive intestinal cells (**c, d**) at day 3 after being treated by sham irradiation + PBS (PBS group),12 Gy abdominal X-ray (IR)+PBS, IR+ Cap@AMF, and IR+SP@AMF (n=6 biologically independent animals). Scale bar =100 μm. The data show means + SD. *P* was calculated using two-tailed t test. *, *P* versus IR+PBS group (*< 0.05, **< 0.01, ***< 0.001, n.s., no significance). Experiment was repeated three times independently with similar results. **e-g.** Pro-inflammatory cytokines including IL-1β (**e**), IL-6 (**f**), and TNF-α (**g**) in the small intestine tissue after different treatments (n=6 biologically independent animals). The data show means + SD. *P* was calculated using two-tailed t test. *, *P* versus IR+PBS group (*< 0.05, **< 0.01, ***< 0.001, n.s., no significance).

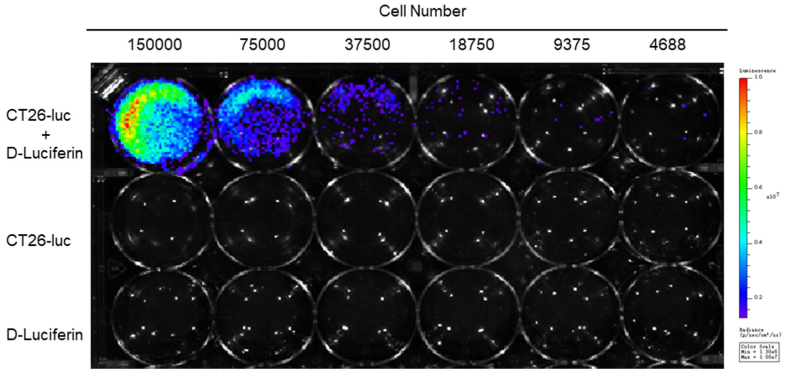

Supplementary Fig. 25. Autofluorescence image of different numbers of CT26-luc cells incubated in a 24-well plate.

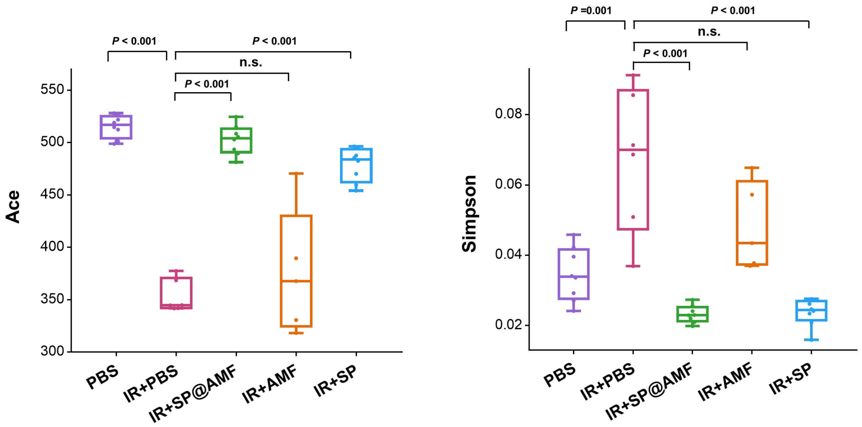

Supplementary Fig. 26. Boxplot of alpha diversity of 16S rRNA gene sequencing of the gut microbiota after different treatments: The index Ace represents the community richness, and the index Simpson represents the community diversity (the higher the Simpson value, the lower the diversity). n=8 for PBS, IR+SP, and IR+SP@AMF; n=6 for IR+PBS; n=5 for IR+AMF (representing biologically independent animals). Results are presented as the boxes' bounds (the 25th to 75th percentile) and lines representing maxima, medians, and minima. *P* was calculated using two-tailed t test.

Supplementary Table 1. Principle of scoring the degree of delayed radiation injury.

Mucosal ulcerations
1 = Small superficial ulcerations
2 = Ulcerations involving more than half of the intestinal circumference
Thickening of serosa
1 = Slight thickening of serosa; hyperplasia of peritoneal
2 = Marked thickening of serosa
3 = Extreme thickening and fibrosis of serosa
Epithelial atypia
1 =Abnormally oriented crypts
2 = Irregular crypt regeneration with ciratypical epithelial cells
3 = Adenocarcinoma
Vascular sclerosis
1 = Slight thickening and hyalinization of vessel wall
2 = Vessel wall double normal thickness; hyalinization and stenosis
3 = Extreme sclerosis with marked stenosis or complete occlusion; fibrinoid necrosis
Intestinal wall fibrosis
1 = Submucosa double normal thickness; broadened and hyalinized collagen fibres
2 = Submucosa three to four times normal thickness; abnormal collagen fibres
3 = Massive fibrosis including muscularis
Lymph congestion
1 = Dilated lymph vessels or cystic collections of lymph
Ileitis cystica profunda
1 = Submucosal glandular inclusions
2 = Submucosal cysts with PolYPoid ekvation of the mucosa
3 = Large cysts extending into the muscularis
