# Supplementary material for: Microalgae-based oral microcarriers for gut microbiota homeostasis and intestinal protection in cancer radiotherapy
Source: Nat Commun. 2022 Mar 17;13:1413. doi: 10.1038/s41467-022-28744-4 (PMC8931093; doi:10.1038/s41467-022-28744-4)
Supplement: Supplementary file 2 — Reporting Summary [file 41467_2022_28744_MOESM2_ESM.pdf]

nature portfolio

Corresponding author(s): Wei Tao, Min Zhou

Last updated by author(s): Jan 16, 2022

Reporting Summary

Nature Portfolio wishes to improve the reproducibility of the work that we publish. This form provides structure for consistency and transparency in reporting. For further information on Nature Portfolio policies, see our Editorial Policies and the Editorial Policy Checklist.

Statistics

For all statistical analyses, confirm that the following items are present in the figure legend, table legend, main text, or Methods section.

n/a	Confirmed	
☐	☒	The exact sample size (*n*) for each experimental group/condition, given as a discrete number and unit of measurement
☐	☒	A statement on whether measurements were taken from distinct samples or whether the same sample was measured repeatedly
☐	☒	The statistical test(s) used AND whether they are one- or two-sided<br>*Only common tests should be described solely by name; describe more complex techniques in the Methods section.*
☐	☒	A description of all covariates tested
☒	☐	A description of any assumptions or corrections, such as tests of normality and adjustment for multiple comparisons
☐	☒	A full description of the statistical parameters including central tendency (e.g. means) or other basic estimates (e.g. regression coefficient) AND variation (e.g. standard deviation) or associated estimates of uncertainty (e.g. confidence intervals)
☐	☒	For null hypothesis testing, the test statistic (e.g. *F*, *t*, *r*) with confidence intervals, effect sizes, degrees of freedom and *P* value noted<br>*Give P values as exact values whenever suitable.*
☒	☐	For Bayesian analysis, information on the choice of priors and Markov chain Monte Carlo settings
☒	☐	For hierarchical and complex designs, identification of the appropriate level for tests and full reporting of outcomes
☒	☐	Estimates of effect sizes (e.g. Cohen's *d*, Pearson's *r*), indicating how they were calculated

Our web collection on statistics for biologists contains articles on many of the points above.

Software and code

Policy information about availability of computer code

Data collection	UV-Vis spectroscopy data was collected by UV-2600 spectrophotometer (Shimadzu, Japan). Fourier transform infrared (FTIR) spectra was detected by FTIR spectra Nicolet NEXUS 470 (Thermo Fisher Scientific, USA). Bright field and fluorescence images were collected by Optical microscope (Zeiss, Germany) or Laser Scanning Confocal Microscopy (Nikon A1, Japan). Field emission scanning electron (SEM) images was collected by SEM microscopy (Hitachi SU-70, Japan). In vivo fluorescence images were collected by an IVIS Lumina LT Series III (Perkin Elmer, USA).
Data analysis	In vivo fluorescence intensity was analyzed by Living Image 4.5 software (Perkin Elmer, USA). The length of small intestinal villus was measured by Image J (1.8.0_112, National Institutes of Health, USA).The LC-MS data was analyzed and output by Agilent Masshunter Workstation (Version B.07.00) software.

For manuscripts utilizing custom algorithms or software that are central to the research but not yet described in published literature, software must be made available to editors and reviewers. We strongly encourage code deposition in a community repository (e.g. GitHub). See the Nature Portfolio guidelines for submitting code & software for further information.

Data

Policy information about availability of data

All manuscripts must include a data availability statement. This statement should provide the following information, where applicable:

- Accession codes, unique identifiers, or web links for publicly available datasets
- A description of any restrictions on data availability
- For clinical datasets or third party data, please ensure that the statement adheres to our policy

The authors declare that all data supporting the results of this study are available from the article and the Supplementary Information. Source data are provided with this paper. Any remaining data are available from the corresponding authors on reasonable request.

Field-specific reporting

Please select the one below that is the best fit for your research. If you are not sure, read the appropriate sections before making your selection.

☒ Life sciences ☐ Behavioural & social sciences ☐ Ecological, evolutionary & environmental sciences

For a reference copy of the document with all sections, see nature.com/documents/nr-reporting-summary-flat.pdf

Life sciences study design

All studies must disclose on these points even when the disclosure is negative.

Sample size	The sample sizes were chosen according to the published works with similar experimental design.
Data exclusions	In 16S rRNA gene sequencing, the data of 2 samples in IR+PBS group and 3 samples in IR+AMF group was excluded because of the failure in the database construction.
Replication	Dara are presented as means and SD of at least 3 independent experiments. All attempts at replication were successful.
Randomization	Mice and cultured cell plates were randomly assigned to different experimental treatments.
Blinding	All data collection and analysis were blinded to group allocation.

Reporting for specific materials, systems and methods

We require information from authors about some types of materials, experimental systems and methods used in many studies. Here, indicate whether each material, system or method listed is relevant to your study. If you are not sure if a list item applies to your research, read the appropriate section before selecting a response.

Materials & experimental systems

n/a	Involved in the study	
☐	☒	Antibodies
☐	☒	Eukaryotic cell lines
☒	☐	Palaeontology and archaeology
☐	☒	Animals and other organisms
☒	☐	Human research participants
☒	☐	Clinical data
☒	☐	Dual use research of concern

Methods

n/a	Involved in the study	
☒	☐	ChIP-seq
☒	☐	Flow cytometry
☒	☐	MRI-based neuroimaging

Antibodies

Antibodies used	For the evaluation of the DNA double strands breaking of the cells after irradiation, γH2AX antibody (Rabbit polyclonal IgG, ab11174, Abcam, Shanghai, China)(1:200 dilution) and secondary antibody (Goat Anti-Rabbit IgG, ab6721, Abcam, Shanghai, China)(1:1000 dilution) were used.
Validation	Abpromise guarantee covers the use of ab11174 in the following tested applications:IHC-P,ICC/IF,WB.

Eukaryotic cell lines

Policy information about cell lines

Cell line source(s)	IEC-6 (ATCC CRL-1592, EK-Bioscience, Shanghai);CT26-luc (Sciencelight Biology, Shanghai);CT26(ATCC# CRL-2638,EK-Bioscience, Shanghai)
Authentication	The used cell lines were morphologically confirmed.
Mycoplasma contamination	No mycoplasma contamination was found.
Commonly misidentified lines (See ICLAC register)	The used cell lines are not listed in ICLAC.

Animals and other organisms

Policy information about studies involving animals; ARRIVE guidelines recommended for reporting animal research

Laboratory animals	Six－week－old female Balb/c nude mice;Six－week－old female Balb/c mice
Wild animals	No wild animal is used.
Field-collected samples	No Field-collected sample is used.
Ethics oversight	All animal experiments were carried out according to the protocols approved by the Institutional Animal Care and Use Committee of Zhejiang University School of Medicine.

Note that full information on the approval of the study protocol must also be provided in the manuscript.
